# Sperm-dependent asexual hybrids determine competition among sexual species

**DOI:** 10.1038/s41598-018-35167-z

**Published:** 2019-01-24

**Authors:** Karel Janko, Jan Eisner, Peter Mikulíček

**Affiliations:** 1 Laboratory of Fish Genetics, Institute of Animal Physiology and Genetics, Academy of Sciences of the Czech Republic, Rumburská 89, 27721 Liběchov, Czech Republic; 2 Department of Biology and Ecology, Faculty of Science, University of Ostrava, Chitussiho 10, 71000 Ostrava, Czech Republic; 3 Department of Mathematics and Biomathematics, Faculty of Science, University of South Bohemia, Branišovská 1760, 37005 České Budějovice, Czech Republic; 4 Department of Zoology, Faculty of Natural Sciences, Comenius University in Bratislava, Mlynská dolina, Ilkovičova 6, 84215 Bratislava, Slovakia

## Abstract

Interspecific competition is a fundamental process affecting community structure and evolution of interacting species. Besides direct competition, this process is also mediated by shared enemies, which can change the outcome of competition dramatically. However, previous studies investigating interactions between competing species and their parasites (parasite-mediated competition) completely overlooked the effect of ‘sperm’ parasites (i.e. sperm-dependent parthenogens or pseudogams) on competition. These organisms originate by interspecific hybridization, produce clonal gametes, but exploit parental species for their own reproduction, being therefore analogous to classical parasites. Here we use the reaction-diffusion model and show that pseudogams alter the outcome of interspecific competition significantly. They may either slow down competitive exclusion of the inferior competitor or even turn the outcome of competition between the species. Asexual organisms may thus have unexpectedly strong impact on community structure, and have more significant evolutionary potential than was previously thought.

## Introduction

The diversity of extant organisms results from interplay of various intra- and interspecific interactions including competition, parasitism, hybridisation. These have been mostly studied on sexually reproducing species due to the vast predomination of sex among Metazoans. However, during the course of evolution the molecular and cytological machinery ensuring sexual reproduction have been modified in numerous ways towards asexuality (e.g.^1^). While the so-called ‘asexual’ organisms proved as suitable models to understand the dis/advantages of sex, their importance remains rather underappreciated and their effect on biological diversity is particularly poorly understood. The collective term ‘asexuality’ encompasses various reproductive modes, but among these pseudogamy, or more specifically sperm-dependent parthenogenesis, occupies a particular position not only for historical reasons (the first documented asexual vertebrate was the psedogamous Amazon Molly fish^2^) but also for evolutionary interest. In this study we show that contrary to expectations, asexual organisms with a sperm-dependent mode of reproduction can affect the diversity of related sexual species through the process analogous to parasite-mediated competition.

**Figure 1:**
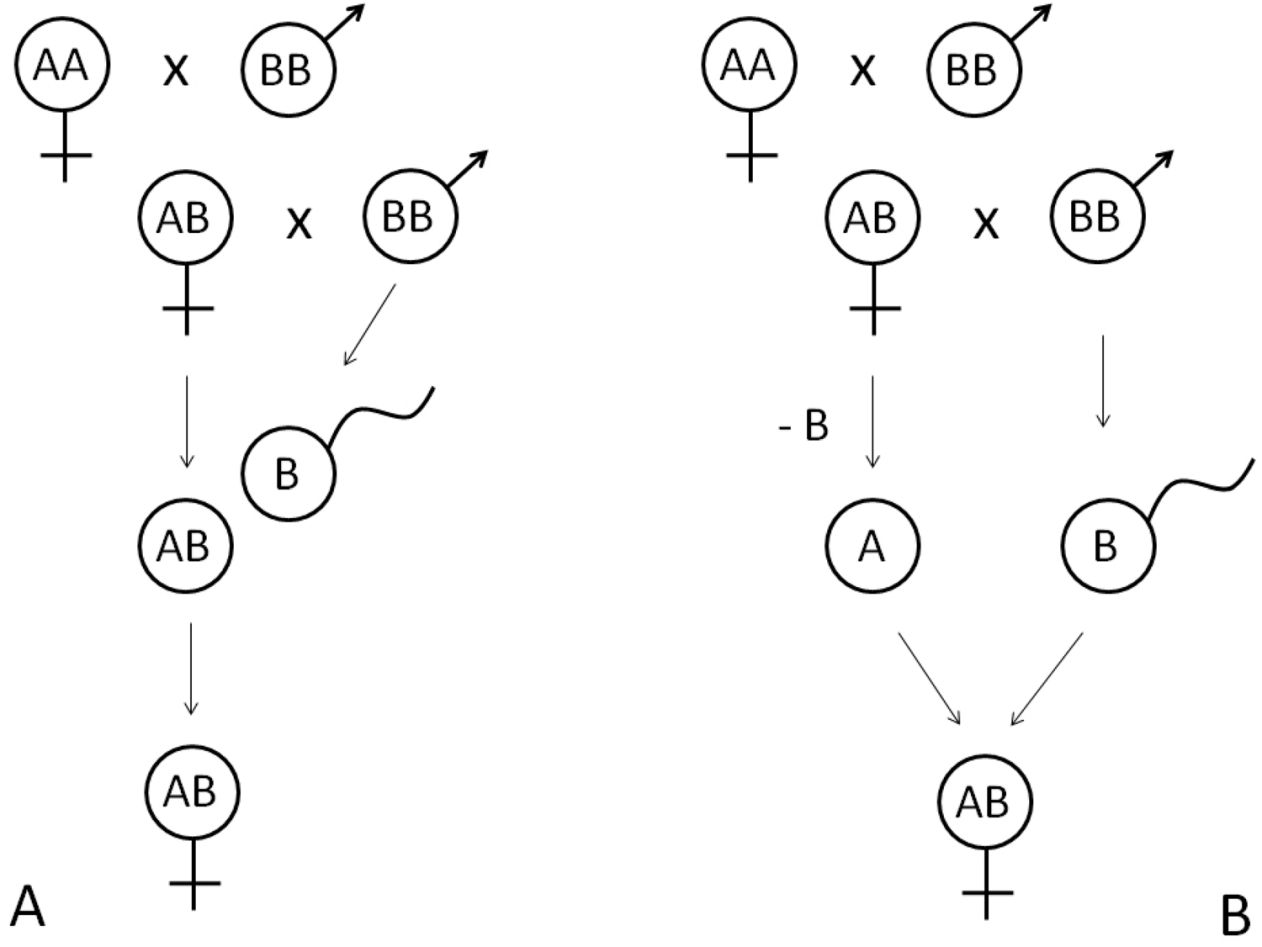
Two major types of pseudogamous reproduction known in animals. In both cases, hybridization between sexual species AA and BB forms a hybrid progeny (AB females in this case). Hybrid females backcross with males of the parental species BB. In gynogenesis (panel A), the sperm only activates the egg without its fertilization, and both parental genomes are clonally inherited. In hybridogenesis (panel B), a hybrid female eliminates one of the parental genomes (−B) and forms clonal eggs, which are fertilized by sperm of a parental species whose genome was eliminated (BB in this case). Gynogenetic and hybridogenetic hybrids ‘steal’ gametes of sexual species for their own reproduction, being often referred as sperm-dependent pseudogams or sexual parasites. Note that in many gynogenetic and hybridogenetic systems, pseudogamous hybrids often simultaneously exploit two or more sexual species for their reproduction.

Pseudogamous taxa generally consist of all-female populations (for exceptions see e.g.^3,4^), which are reproductively dependent on the sperm of coexisting sexual males (usually belonging to their ancestral and/or other reproductively compatible sexual species^5^), since the development of a clonal ovum requires activation or fertilization by a sperm (Fig. 1). Pseudogams thus represent a special puzzle in evolution as they exhibit the disadvantages of both sexual and asexual reproductive modes^6^, i.e. they lack effective recombination but simultaneously depend on obtaining a mating partner. Due to these properties, pseudogamous systems have commonly been regarded as entities with a rather ephemeral evolutionary potential^7^.

While pseudogamous asexuals rely on the availability of sperm, from the point of view of their host this represents a wastage since the genome of the sperm is lost either immediately after egg activation (in gynogenetic pseudogamy) or in the next generation (in hybridogenetic pseudogamy; Fig. 1; e.g.^6^). Such an asymmetrical relationship leads to the perception of sperm-dependent parthenogens as ‘sperm parasites’^8^ or ‘sexual parasites’^9^.

There are indeed many analogies between the pseudogams and the parasites in the literal meaning of the word. For example, true parasites tend to reduce the reproductive success of their hosts (e.g.^10–13^), tend to reduce the hosts’ population density^14^ and affect the hosts’ spatial structure and dispersal^15,16^. Similarly, pseudogams reduce the host’s reproductive success by ‘stealing’ its gametes, decrease host density by utilizing available resources^17,18^ and may even reduce the host’s spatial expansion^19^.

It is also noteworthy that sexual sperm-donors, analogously to the hosts of ‘classical’ parasites, employ various mechanisms to increase their ‘immunity’. Such countermeasures against pseudogamy include niche segregation^20^, spatial differentiation^21^ and behavioural adaptation^22^. In particular, the mating preference of sexual males for their own females, might represent a prominent mechanism for stabilizing the coexistence of sexuals and pseudogams^23–25^.

Nevertheless, while the analogies between sexual-pseudogamous and host-parasite systems have increased our understanding in many ways^26^, one crucial aspect has been omitted, although its consideration may conceptually shift our perception of sexual-pseudogamous systems. Specifically, the theoretical models have so far only considered bilateral interactions between sexual and asexual population components despite most pseudogams being hybrids of two or more of the sexual species with which they interact. Such parental species are often in competitive interactions with each other and possess distribution ranges that overlap in relatively narrow (e.g. *Bacillus*^27^; *Ambystoma*^28^; *Poecilia*^23^; *Poeciliopsis*^29^; *Cobitis*^5^) or extensive (e.g. *Pelophylax*^30^; *Chrosomus*^31^) hybrid zones. Hybrid zones in general have attracted considerable research interest as evolutionary laboratories may also impose specific effects on interspecific competition^32^. Unlike ‘classical’ hybrids with Mendelian inheritance however, the asexual hybrids emerging in such zones maintain their genetic integrity and often expand into allopatric parts of both parental ranges^5^.

It follows that a given pseudogamous form arisen from the hybridization of two sexual species often backcrosses with two or even more sexual hosts simultaneously (rev. in^5^). This property reminds true for parasites that commonly affect more than one host species too. In community ecology, the competition mediated by parasites (also called ‘apparent’ competition or ‘parasitic arbitrage’) is considered of major importance, e.g.^33,34^. This type of indirect interaction is defined as a negative effect of one species on the abundance or population growth of another species, mediated by shared natural enemies, and it may occur regardless of the two species competing directly for resources^35^. Higher resistance to a shared parasite in one species gives it an important advantage even if it is an otherwise inferior competitor^36,37^.

In this paper we argue that our understanding of the evolutionary potential of pseudogamy would benefit from a conceptual shift similar to the current one in studies of species interactions, i.e. the move from pairwise models to competitive network analyses^38^. Specifically, using spatial population models based on Lotka-Volterra equations we demonstrate the benefit of viewing pseudogams as a sort of shared parasites potentially mediating the competition between two or more sexual species. We show that inclusion of multilateral interactions between the pseudogams and their individual host species as well as among the sexual hosts themselves reveals qualitatively novel and potentially strong mechanism via which asexuals may alter the ecology and evolution of sexual species. Given that asexual organisms are found in many animal and plant taxonomic groups with new cases being discovered almost every year, we believe our finding will open new conceptual look at their importance in evolution.

## Results

### The model description and simulation strategy

#### Spatial vs. non-spatial aspects of the model

To investigate the multilateral interactions among sexual species and their pseudogamous hybrids, we use a model based on the Lotka-Volterra equations, which have commonly been applied to study both interspecific competition and sexual-pseudogamous systems^39–41^. Our model incorporates two sexual species, both with a 1:1 sex ratio (hereafter also referred to as species *S*_1_ and *S*_2_) that compete for common resources and hybridize upon contact to produce pseudogamous hybrid females (hereafter *H*). Hybrid females are reproductively dependent on males of either *S*_1_ or *S*_2_ species. The model is described in the Methods section in detail and we briefly present it here.

We first considered the competition between the three forms (*S*_1_, *S*_2_, *H*) across time. This situation is described by three ordinary differential equations (ODE, see Methods) whose parameters are described below. Since the interactions among species take place not only in time but also in space, we subsequently added the diffusion of individuals spatially and studied the system using partial differential equations (PDE, the full model). In the latter case, species *S*_1_ and *S*_2_ were introduced at opposite edges of one-dimensional space and were allowed to expand and make reproductive and competitive contact, as if for example two species of fish colonizing a stream from opposing ends. After hybridization, hybrid females subsequently invaded the territories of both species competing for resources and sperm.

#### Parameters of competition and mate choice

Both ODE and PDE systems involve the following key parameters: Parameters *α*_*ij*_ represent the intraspecific (*i* = *j*) and interspecific (*i* ≠ *j*) competition coefficients (index values *i*, *j* = 1, 2, 3 refer to *S*_1_, *S*_2_ or *H*, respectively, see Eqn. (1) in Methods). Parameters *A* and *B* describe the mating preferences of *S*_1_ or *S*_2_ sexual males for allospecific females relative to their own females (Eqs (2)–(5) in Methods; for simplicity, we assumed that males have the same preference for all allospecific females i.e. the heterospecific sexual and hybrid asexual ones). Modelled scenarios assume that *S*_1_ may either be competitively equal or superior to *S*_2_. Therefore, unless stated otherwise, we will use terms ‘superior’ and ‘inferior’ competitor as synonyms for *S*_1_ and *S*_2_ species, respectively.

#### Population birth functions

To link the model to the biological reality and to minimize the risk that the revealed pattern would be specific only to a particular model design, we took into account the effects of various biologically relevant scenarios, which are implemented into our equations via specific birth functions, types of competitive and mate choice asymmetries as well as types of modelled hybrids.

Birth functions were adapted from^42^ and describe the growth of populations conditional on the probabilities of the formation of inter- and intraspecific mating couples of males and females while taking into account the mating preferences. However, in contrast to^42^, we assume that males, rather than females are the choosy sex. Our simulations thus considered four types of population growth functions with respect to sex- and gamete-limitation, i.e. Eqs (2)–(5). Eqn. (2) assumes that population growth is proportional to the product of encounters of appropriate males with appropriate females divided by total amount of females available for given males (i.e. the sum of all conspecific and the fraction of heterospecific females given males’ mating preferences).

Although relatively simple to calculate, the birth functions in Eqn. (2) have the undesirable property of strong male-limitation as they induce steep decrease of *S*_1_ or *S*_2_ species’ population growth rates when proportion of heterospecific females increases since rapidly decreasing proportion of males would remain available to form homospecific pairs. To reduce such effect of male dominance, we also performed the simulation with implemented formulas using the harmonic mean (Eqn. (3)), which reduces such effect and assumes intermediate dominance (see e.g.^43^); if females are rare, the birth function tends towards female dominance while if males are rare, it tends to male-dominance.

Both aforementioned birth functions (Eqs (2) and (3)) assume that both sexes have identical efficiency of gamete production and that females and males may reproduce repeatedly. To tackle the problem of stronger female gamete-limitation, we further introduced two variants of these birth functions, i.e. Eqs (4) and (5), which assumed that females that have mated with any male are unavailable to form next mating pair. This assumption has been modelled via introduction of cross-link terms into original Eqs (2) and (3), which reduce the numbers of available females.

#### Effects of asymmetries between species, asexuality and hybrid origin

The main goal of the simulation was to investigate the effect of mating *asymmetries in the ‘resistance’ of both sexual species to shared sperm-parasites*. We studied two sorts of such asymmetries. First, we assumed that species *S*_1_ and *S*_2_ differed in male mating preferences (*asymmetry type 1*–we kept *A* constant and varied *B* from 1 to 0.1). Second, we proposed that the presence of pseudogams exerted asymmetrical competitive pressure on both sexual species (*asymmetry type 2*) keeping *α*_13_ constant and varying *α*_23_ (from 0.2 to 2).

One additional mechanism also had to be involved in our models due to the specific properties of asexual organisms. Specifically, when sexual and asexual counterparts are equal in all parameters except the reproductive mode, asexuals should outcompete sexual species due to their intrinsic reproductive advantage, which would result in the collapse of the whole system because pseudogams would be depleted from the sperm resource. To avoid such a situation, some sort of mechanism stabilizing the sexual-asexual coexistence has to be implemented. We included two distinct types of such *stabilizing mechanisms (SMs)* in our model^40^ in order to test the stability of observed patterns against different assumptions (see the Methods section for details). The first *(SM1)* follows that asexuals are limited by a higher intensity of intraspecific competition due to stronger competition of self-identical asexual individuals as proposed by^44^. We therefore used higher *α*_33_ values than *α*_11_ and *α*_22_. The second *stabilizing mechanism (SM2)* assumes the role of mate-recognition. We therefore set the asexuals identical to sexuals in the intraspecific competition coefficients, but fixed the parameter *A* = 0.5 to counterbalance the two-fold reproductive advantage of pseudogamy (the values of *B* varied as we investigated their influence on the model behavior; see above).

Subsequently, we also must take into account the fact that asexuals are hybrids, which may invoke specific properties as compared to their parental species. Specifically, to evaluate the *effect of hybrid origin*, our model considered three possible types of hybrids that differ in their competitive strength relative to the parental species. We kept coefficient *α*_12_ ∈ (0; 1] as the main ‘control’ parameter of relative strengths for all three hybrid types. *Hybrid type 1* (expression dominance of *S*_1_ species): competitive strength of hybrids was set equal to the superior sexual competitor (*α*_32_ = *α*_12_, inferior sexuals *S*_2_ have the same influence on hybrids as on superior sexuals *S*_1_); *Hybrid type 2* (expression dominance of *S*_2_ species): competitive strength of hybrids was set equal to inferior competitor (*α*_13_ = *α*_12_, hybrids *H* and inferior sexuals *S*_2_ have the same influence on superior sexuals *S*_1_); *Hybrid type 3* (additive expression): competitive strength of hybrids was set to the geometric average of both sexual species (${\alpha }_{13}={\alpha }_{32}=\sqrt{{\alpha }_{12}}$, which means that the influence of inferior species *S*_2_ on hybrids is proportionally the same as the influence of hybrids on superior species *S*_1_, i.e. the ratio of competitive strengths of *S*_1_ versus *H* is the same as that of *H* versus *S*_2_; roughly speaking, *α*_13_ = *S*_1_/*H* = *H*/*S*_2_ = *α*_32_ and *α*_12_ = *S*_1_/*S*_2_ = (*S*_1_/*H*):(*S*_2_/*H*) = *α*_13_/(1/*α*_32_) = *α*_13_*α*_32_).

Finally, our model also acknowledged the debate about how often may interspecific hybridization give rise to successful clones, which is ongoing for at least 100 years since^45^. Some species combinations generated pseudogamy only upon unique evolutionary events (e.g.^46^), while others do so rather frequently (e.g.^47^). Therefore, we also simulated the case when only small fraction of interspecific crosses gave rise to successful pseudogams which subsequently mate with the parental species. In this case, we assumed that only 1% of ${S}_{1}^{\male}\times {S}_{2}^{\female}$ or ${S}_{2}^{\male}\times {S}_{1}^{\female}$ pairs produce clonal progeny, but all inseminations of *H*^♀^-s lead to viable progeny).

#### Evaluating the role of pseudogams–simulation strategy

Having detailed the model and its parameters, we now describe the strategy how we evaluated the implicit effects of pseudogamy on interspecific competition. We compare the full model of two hybridizing species producing pseudogamous hybrids (called run type 1 or RT1 in the following text) with two reference runs simulating the situations when hybridization does not induce pseudogamy. Specifically, we compared run type 1 with the situation when both sexual species compete but do not hybridize (i.e. the pure-competition model where *A* = *B* = 0; we refer to such a simulation as run type 3 or RT3). Next, we also compared run type 1 with the situation when both sexual species simultaneously compete and cross-bred but do not produce asexual hybrids (we call such a simulation as run type 2 or RT2 in the following text). Run type 2 thus mimics the classical hybrid zone and was designed so that the variable *H* in the numerators of the *β*_3_-equation in Eqs (2)–(5), respectively, equalled zero, i.e. *S*_1_ and *S*_2_ species do hybridize but produce only sterile progeny. This is analogous to a case of ‘classical’ hybrid zone with strong under-dominance. We selected this design of run type 2 for two reasons. First, the classical equations used by Barton^32^ to study the behavior of hybrid zones model allele frequencies rather than distinct biotypes as in our case. Therefore, Barton’s equations may implicitly incorporate any type of backcross individual, while our model involves a separate equation for the stable hybrid form (asexuals neither recombine nor segregate). Second, it is known that extreme hybrid under-dominance maximally attenuates the rate at which the contact zone moves^32^ compared to the pure-competition model, which justifies our simplifying assumption. This is because if pseudogams were found to affect the hybrid zone movement more significantly than sexual hybrids (run type 2), they would then logically have a stronger effect than any other type of more fit sexual hybrids (i.e. those with weaker under-dominance).

### Simulation results: the effect of pseudogamy on interspecific competition

**Figure 2:**
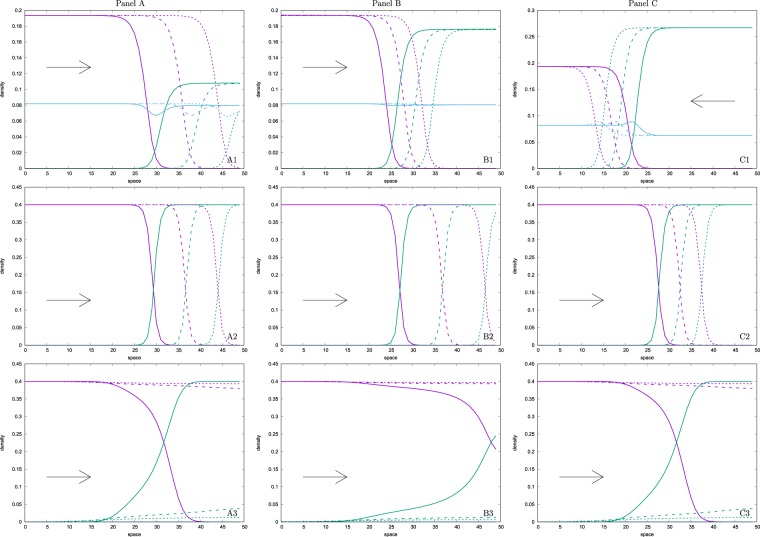
Influence of hybrids on apparent competition of sexual species. On each panel we observe space profiles and movement of travelling waves of densities of the three population components (i.e. *S*_1_ species (purple lines), *S*_2_ species (green lines) and of a hybrid (*H*) population (light blue lines), respectively). The sections are taken at three time slices (*t*_1_, *t*_2_ and *t*_3_ denoted by a full, dashed and dotted, respectively, line type) that are homologous among panels. On the upper row, we demonstrate run type 1 (RT1, i.e. the model of two hybridizing species producing pseudogamous hybrids), middle row run type 2 (RT2, i.e. the model of two hybridizing species producing sexual hybrids) and lower row run type 3 (RT3, i.e. the model when two sexual species compete but do not hybridize). Panel A demonstrates the situation when the presence of pseudogamous hybrids hurts the weaker competitor, which dies out more rapidly in run type 1 than in run type 2. On panel B we see a scenario when hybrids help the weaker competitor, which dies out more slowly in run type 1 than in run types 2 and 3. On panel C we demonstrate situation when the presence of pseudogams reverses the interspecific competition so that the weaker competitor wins and replaces the stronger one in RT1 although it loses in RT2 and RT3. Growth functions *β*_*i*_ are of the form Eqn. (3), *Stabilizing Mechanism SM1* (*α*_33_ = 5) is applied, and we assume *hybrids type 3* (they are competitively in-between the superior and the inferior competitor). Other parameters are *α*_12_ = 0.5, *B* = 1 (panel A), *B* = 0.88 (panel B), *B* = 0.7 (panel C), cf. the middle column on Supplementary Figure SF11.

We observed in general three types of results, which are shown on respective panels of the Fig. 2. In the first type, the pseudogamous hybrids had a negative effect on the weaker competitor, which died out sooner in the full model (run type 1) than in ‘classical’ hybridization run type 2 (Fig. 2, panel A), In the second type of result, pseudogams had a positive influence since the extinction of the weaker competitor took more time in run type 1 than in run type 2 (Fig. 2, panel B). Finally, in the third type of result, pseudogams even reversed the competition result, where the weaker competitor survives and the stronger one is out-competed (Fig. 2, panel C).

#### Ordinary differential equations

**Figure 3:**
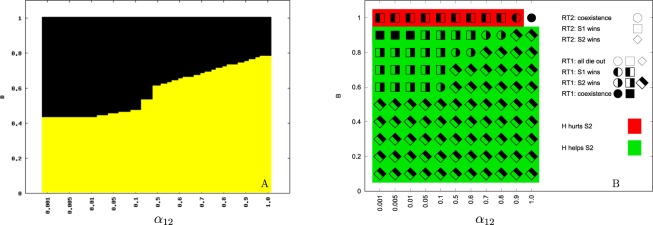
Qualitative comparisons of simulations of the ordinary differential equations (ODE) model without the space dimension (panel A), and of the model of partial differential equations (PDE) with diffusion in space (panel B). We assume *asymmetry type 1* between sexual populations (species *S*_1_ and *S*_2_ differed in male mating preferences) and *hybrids type 1* (hybrids are competitively equal to the superior competitor). *Stabilizing Mechanism SM1* (*α*_33_ = 15) is applied, growth functions *β*_*i*_ are of the form Eqn. (2). Mate choice parameter *A* = 1, *B* varies along the *y*-axis. All *α*_*ij*_ = 1 except *α*_12_–the competitive asymmetry between *S*_1_ and *S*_2_ species, which is varied along the *x*-axis. On panel A there are 33 (horizontally) times 100 (vertically) calculated grids visualized. Yellow fields demonstrate the domains of parameter values where the inferior species *S*_2_ survives and the superior species *S*_1_ is out-competed. In black, there are domains where *S*_2_ dies out. On panel B we compare the results of model behavior assuming hybridization producing pseudogamous hybrids (run type 1, RT1) with those where hybrids do not reproduce asexually (i.e. hybrid zone formed with strong under-dominance; run type 2, RT2). The parameter space is divided into 11×10 grids. A symbol at each grid demonstrates the simulation result. Symbols’ shape indicates the result of the reference run type 2 (circles = coexistence of both hybridizing competitors; square = replacement of the inferior competitor; diamond = replacement of the superior competitor). Symbols’ infill indicates the result of run type 1 (black filling of the entire symbol = coexistence of both competitors, filling of only left or right parts = the outcompetition of the inferior or superior species, respectively). Background color allows the comparison between run types 1 and 2. Red highlights the cases when replacement time of the inferior species was shorter in run type 1 than in run type 2 (i.e. when the pseudogams boosted the competitive exclusion and hence their presence hurts the weaker competitor). Green highlights the cases when the replacement time of the inferior species was longer in run type 1 than in run type 2 or even the inferior species replaced the superior one (i.e. when the pseudogams helped enough the inferior competitor).

Simulations of the ODE system without the space dimension showed that hybrids are able to change the expected result of the competition between their parental sexual species. While in the pure competition model the inferior competitor is always outcompeted, in the model with pseudogamous hybrids this is not always the case. Namely, the Fig. 3, panel A demonstrates that the inferior competitor can out-compete the superior one if it is less sensitive to the pseudogamous parasites, for example in the situation where its males have stronger mate choice preferences for its own females. The parameter spaces allowing the out-competition of the stronger competitor by the weaker one are shown in detail in Supplementary Figures SF1–SF8 for various scenarios of stabilizing mechanisms and hybrid types.

#### Partial differential equations

In the system of PDE that takes into account the spatial context, we also confirmed that the presence of pseudogamous hybrids had a significant effect on the interspecific competition of sexual species (Fig. 3, panel B).

Pure competition model (RT3): To understand the specific effects of pseudogamy on interspecific competition, we first describe the outcomes of the reference simulations. Run type 3, assuming the pure competition model, provided an unsurprising result; the superior competitor always replaced the inferior one (Fig. 2, bottom row). The replacement was realized via a travelling wave with the speed proportional to the differences in competitive strength between both competitors.

Model of hybrid zone (RT2) and the effects of mate choice: When we modelled the case of the ‘classical’ hybrid zone and included the reproductive interactions between both species leading to sterile hybrids (run type 2), we observed qualitative as well as quantitative changes compared to run type 3. Although the superior competitor generally also tended to replace the inferior one, hybridization notably decreased the rate of replacement compared to the pure-competition model (run type 3) (see Fig. 2, middle vs. bottom rows, and Supplementary Figures SF9, SF11, SF13, SF15 and middle rows of Supplementary Figures SF10, SF12, SF14, SF16 i.e. cases when *B* = *A* or *α*_23_ = *α*_13_). In fact, when competitive differences among species were subtle (*α*_12_/*α*_21_ > 0.8), hybridization even apparently stabilized the coexistence of both sexual competitors (Fig. 2, middle row, Fig. 3, panel B, Supplementary Figures SF9–SF16, cases when *A* = *B* or *α*_13_ = *α*_23_). In these cases we observed no significant area shift until the end of the simulation run as the speed of the travelling wave apparently approached zero.

We also noticed that the spatial overlap between competing species was narrower in the case of a hybrid zone (run type 2) than in the pure-competition model (run type 3; see Fig. 2, middle vs. bottom rows), which is consistent with the results of^32^. We further observed that the speed and sometimes even the direction of the hybrid zone propagation was dependent on the difference between mate recognition ability of both species or their relative competition intensity with hybrids (i.e. on the proportion of *A* and *B* or *α*_13_ and *α*_23_ values, respectively). In general, for given values of *α*_12_ and *α*_21_, better mate recognition of the inferior species (i.e. $B < A$) delayed its replacement time because it reproduced more effectively while investing less effort in mating with the superior competitor. In extreme cases when $B\ll A$, coexistence was observed or the inferior competitor even replaced the superior one due to its better mate recognition (Fig. 3, panel B, Supplementary Figures SF9–SF12). Similarly, when the inferior competitor was less affected by competing hybrids in the hybrid zone (i.e. ${\alpha }_{23}\ll {\alpha }_{13}$), it could replace the superior competitor.

Model of pseudogamy (RT1): The aforementioned analysis of run type 2 demonstrates that hybridization in general has a major impact on interspecific competition. In the next section, we show that involvement of sperm-dependent asexuality (run type 1) has an even greater effect on both the quantitative as well as the qualitative outcome of interspecific competition. Without specific mechanisms stabilizing the coexistence of pseudogams with their sexual counterparts, the pseudogamous hybrids tended to outcompete both parental species, due to their higher intrinsic growth rate. The simulations then resulted in the collapse of the whole system. However, when either type of stabilizing mechanisms was enforced (i.e. SM1 or SM2), we observed that asexuals were able to coexist with one or both sexual species (depending on the result of competition) over a large parameter space. The ultimate ratio of sexual and asexual counterparts in mixed populations depended on the relative strength of mate choice or intraspecific competitive asymmetry. In other words, the more intensive the intraspecific competition within a pseudogamous hybrid, the lower the proportion of such asexuals in the entire population. Similarly, the increasing ability to discriminate among mating partners of given species (*S*_1_ or *S*_2_) resulted in an increasing proportion of that species in the mixed sexual-pseudogamous population.

The presence of pseudogams systematically affected the result of interspecific competition between sexual species. Higher resistance of the inferior competitor to pseudogams (i.e. either *B* < *A* or *α*_13_ > *α*_23_) turned into its advantage so that for a given ratio of *α*_12_/*α*_21_ interspecific competition coefficients, the replacement time was longer compared to equivalent run types 2 and 3 (Fig. 2, panel B, Fig. 3, panel B, and Supplementary Figures SF9–SF16). Further increase of the resistance (when $B\ll A$ or ${\alpha }_{13}\gg {\alpha }_{23}$) led to the coexistence of both competitors or even to the outcompetition of the superior competitor (Fig. 2, panel C, Fig. 3, panel B and Supplementary Figures SF9–SF16). This effect of pseudogams was caused by the fact that asexuals tended to reduce the growth rate of the superior competitor due to its higher reproductive investment into pseudogamous females. In extreme cases when values of *B* were too low, the hybrid population only persisted in the region occupied by the superior competitor since too strong mate recognition of the second species prevented the pseudogams’ spread and survival in its region.

On the other hand, when both sexual species had a similar level of resistance to pseudogams (i.e. *A*≈*B* or *α*_13_≈*α*_23_), or even the superior species was more resistant ($A < B$ or ${\alpha }_{13} < {\alpha }_{23}$), the superior competitor replaced the inferior competitor with a greater speed than in run type 2 (Supplementary Figures SF9–SF16). This is because hybrids entered the area of the inferior competitor and decreased its growth rate beyond the invading wave of the superior competitor and hence facilitated its invasion.

The aforementioned results and trends were valid for all types of modelled hybrids. The differences among simulation runs with pseudogams of *hybrid types 1–3* were only quantitative but not qualitative–the type of hybrid assumed affected only the parameter values for which different types of results were observed.

The observed trends were insensitive to perturbations of initial conditions even if the initial seed values for *S*_1_ and *S*_2_ species were non-symmetrical, i.e. when one species has been introduced in higher initial densities. The only difference was in the size of the domain occupied by each sexual species but final levels of population densities as well as the competition results were independent of the initial conditions.

Also, no change was observed even when the production of pseudogams from interspecific crosses has been decreased to only 1%.

## Discussion

Interspecific competition is one of the major forces in shaping global biodiversity. When competing species cannot sufficiently differentiate their niches, the inferior competitor is expected to be outcompeted by the superior one. This expectation is indeed reflected in our models assuming pure competition by the basic observation that the inferior competitor always lost (bottom row on Fig. 2). Nonetheless, competition in nature is more complex and generally involves diverse competitive networks rather than only pairs of competing species^38,48^. As we show in this paper, the inclusion of shared pseudogamous parasites may considerably change the outcome of interspecific competition.

In systems where sexual and sperm-dependent asexual forms interact, the sexual species, which is more resistant to pseudogams (e.g. the one that better discriminates its own mating partners, or who is less affected by hybrids’ pressure), gains the advantage in the interspecific competition even if it normally appears as the inferior competitor. When competitive asymmetries between sexual species are weak, even slightly better mate discrimination or slightly lower sensitivity to hybrids may decisively change the result of interspecific competition in favour of the inferior competitor. For certain parameter-space, the pseudogams may even ultimately mediate the outcompetition of the superior competitor by the inferior one (Supplementary Figures SF1–SF16).

### Competition on a spatial scale and the role of hybridization

Since the interactions among species take place not only in time but also in space, the effect of spatial distribution on competition mediated by pseudogamous hybrids should be investigated. It is generally expected that the stronger competitor disperses at the expense of the inferior one. Fisher and Kolmogoroff^49,50^ derived the minimal speed of the travelling wave front of the superior invader on homogeneous space at ${V}_{{\rm{\min }}}=2\sqrt{kS}$; where *k* is the diffusion coefficient and *S* is the selective advantage of the superior species. Murray^41^ showed that analogous expression can be derived for competitive asymmetry in terms of the parameters of the Lotka-Volterra model used in our equations.

Nonetheless, the dynamics of interspecific competition may be dramatically modified if both competitors hybridize^32^. Indeed, it is often the case that competing species maintain the ability to interbreed but the fitness of their hybrids tends to decrease over evolutionary time (e.g.^51^). When such under-dominance occurs, a sort of barrier may become established between hybridizing competitors^32^ and the advance rate of the superior competitor is then smaller compared to the pure-competition model. Expressing the fitness of competing species *P* and *Q* as 1 + 2*S* and 1, respectively (see Barton’s model^32^), but assuming a lower than average fitness of the *PQ* heterozygote (1 + *S* − *s*), the advance rate of superior competitor decreased to an equilibrium value either equaling $2\sqrt{k(S-s)}$ for *S* > 2*s*, or $S\mathrm{/2}\sqrt{2m/s}$ for $S < 2s$ (i.e. when under-dominance is strong). Therefore, the hybrid zones tend to delay the exclusion of the inferior competitor.

Our results (run type 2) agreed with the aforementioned predictions but we also found that the evolution of a hybrid zone may further depend on mate choice. A higher ability of the inferior competitor to discriminate mating partners may slow or halt the hybrid zone advance and extreme mate choice asymmetries may even reverse the direction of hybrid zone propagation leading to the outcompetition of the superior competitor. Although mechanisms affecting the stability or movement of hybrid zones have been intensively studied (e.g.^52^), we are not aware of any theoretical analyses modelling the effect of mating asymmetries on the movement of the ‘classical’ hybrid zone. However, these results certainly warrant further attention since there are empirical cases demonstrating that asymmetries in mating preferences may affect both the rate and direction of hybrid zone movement^53,54^, which is in line with our model.

### The role of pseudogams in the interspecific competition

While hybridization between competitors significantly affects the competitive outcome we found that pseudogamous reproduction of hybrids induces a specific and qualitatively distinct effect on interspecific competition that has not yet been described. Pseudogams originating in the hybrid zones can expand over allopatric parts of the parental ranges^5^, negatively affecting the host population densities even beyond the hybrid zone (e.g.^17^). When both sexual competitors differ in sensitivity to shared pseudogamous hybrids, pseudogams may become a biological weapon in the hands (or fins, in the case of fish) of the more resistant sexual species. Specifically, population densities in mixed sexual-asexual complexes are higher for the more resistant species (e.g. the one that better discriminates its own mating partners, or who is less affected by hybrid pressure, see also^17^). This consequently modifies the result of interspecific competition. If the inferior competitor was more resistant, then, depending on the level of asymmetry in resistance, pseudogams would increase its chances in interspecific competition by either attenuating the invasion rate of the superior competitor, halting the invading wave or even helping to replace the superior one (Supplementary Figures SF9–SF16).

To understand the specific effects of pseudogamy on interspecific competition, we compared the full model simulating the interactions of two species and pseudogamous hybrids (run type 1) with travelling wave propagation under the pure competition model (no hybridization between sexual competitors; run type 3), and with the movement of the ‘classical’ hybrid zone under strong under-dominance (run type 2). All relevant parameters (i.e. the competitive and mate preference asymmetries between sexual species) remained equal between run types except that we minimized the fitness of hybrids in run type 2, (please note that such a parameter setting mimics the extreme under-dominance in the hybrid zone and has been shown to maximally attenuate the rate at which the contact zone moves^32^). Yet, even in such comparisons, the effect of pseudogams on wave advancement was stronger in the full model (run type 1) compared to the ‘classical’ hybrid zone (run type 2). For example, we observed, as expected, that the invasion of the superior competitor was always slower in run type 2 (with a ‘classical’ hybrid zone) than in the pure-competition model (run type 3). However, under parameter values where competitive and mate preference asymmetries permitted the slow advance of the ‘classical’ hybrid zone in the direction of an expanding superior competitor, hybrid pseudogamy already caused stagnation or even the replacement of the superior competitor with an inferior one in the full run type 1 model (Supplementary Figures SF9–SF16).

In cases when sensitivity to pseudogams was similar for both species or even the superior competitor was more resistant, pseudogams catalyzed a faster exclusion of the inferior competitor compared to run type 2 (Supplementary Figure SF9–SF16). However, we emphasize that for such a particular setting run type 2 does not offer an exact comparison with the scenario with pseudogams, because run type 1 assumes the relatively high fitness of pseudogamous hybrids.

Given that two or more sexual species are often exploited as hosts by the same pseudogamous hybrid form^5^, they may serve as the reservoir of a shared enemy or disease. This makes sexual-pseudogamous systems analogous to systems with apparent competition, which is considered to be one of the most important factors affecting the structure of entire ecosystems (e.g.^55,56^) as well as human societies^57^. Shared disease has been shown to attenuate the advance rate of a superior competitor or cause its replacement by an inferior competitor if it suffers less from the disease^34,58,59^. Our simulations show that sperm-dependent parthenogens may have analogous effects on interspecific competition as shared parasites or pathogens.

Obviously, the sexual-pseudogamous interactions are complex and other scenarios may be quite important for sexual-asexual interactions or hybrid zone propagation, such as the intraspecific variation in male mate choice^24^, spatial heterogeneity^32^, and the outcomes of interspecific competition other than competitive exclusion (niche segregation, local extinctions etc). In any case, incorporation of interactions between parental species significantly increased the complexity of the analyzed sexual-pseudogamous systems and the revealed patterns were robust to various assumptions about the nature of such systems (i.e. frequency of asexual formation, various types of population birth functions, hybrids or stabilizing mechanisms did not affect the results qualitatively).

## Conclusion

The crucial role of parasites on ecosystem diversity and interspecific competition was revealed long ago. On the other hand, sperm-parasites have mostly been viewed as forms with limited evolutionary potential although increasing evidence has been gathered showing that pseudogams may have longer evolutionary life-span than expected^60^ and affect the local (e.g.^6,17^) as well as large-scale^19,61^ diversity patterns of their sperm hosts. Our study uncovered the unexpectedly profound effect of pseudogams on the diversity of sexual species.

Although pseudogamy is rare among animals^6,8,62^, we argue that revealed process may play an important role in the biodiversity since the real incidence of pseudogamous asexuals may be considerably higher than is currently assumed. For example, it is likely that many pseudogamous taxa remain undetected due to their cryptic nature^63^ and new cases are being discovered recently even among commercially exploited species^64^. Moreover, hybrid asexuality appears as an inherent stage of the speciation process when aberrations in meiosis leading to hybrid asexuality tend to evolve earlier during the species diversification, than other classical forms of reproductive isolation mechanisms such as hybrid sterility or inviability^47^. Hybrid asexuality may thus at least temporarily evolve more often than was commonly believed. Given that such hybrid forms often simultaneously exploit several sexual species^5^), the evolution and ecology of various species might have been affected by the hereby described mechanism of competition mediated by a sperm-parasite. This suggests that although individual pseudogamous clones tend to be short-lived as predicted by various theories, their evolutionary significance may by far exceed their lifespan and their effect on the diversity and distribution of sexual species may be long-lasting.

## Methods

### Equations

In this paper, we investigate the spatiotemporal evolution of a sexual-asexual complex and model the competition and hybridization of two sexual species and their pseudogamous hybrids. Both sexual species (referred to as *S*_1_ and *S*_2_) have an equal ratio of males and females. Therefore, if the population density of the first species equals *S*_1_, then the number of males (and females) of that species always equals *S*_1_/2. For better orientation, we always include the symbol ♂ and ♀ in the specific ratios to make it clear which equation member indicates to males and females. Sexual species *S*_1_ and *S*_2_ hybridize when in contact, which leads to the production of pseudogamous hybrid females (*H*) that are reproductively dependent on males of either *S*_1_ or *S*_2_.

The density dynamics of the three forms are controlled by the reaction term stemming from the formulas of Schley *et al*.^40^ who used Lotka-Volterra (LV) models to study the density-dependent dynamics of animal populations with sperm-dependent parthenogenesis. Here, we extended Schley’s two-population response functions to incorporate three competing populations (*S*_1_, *S*_2_, *H*) with the response functions *f*_*i*_, *i* = 1, 2, 3, having the form

1 $$\begin{array}{l}{f}_{1}({S}_{1},{S}_{2},H)={\beta }_{1}({S}_{1},{S}_{2},H)-({\alpha }_{11}{S}_{1}+{\alpha }_{12}{S}_{2}+{\alpha }_{13}H){S}_{1}-{\mu }_{1}{S}_{1};\\ {f}_{2}({S}_{1},{S}_{2},H)={\beta }_{2}({S}_{1},{S}_{2},H)-({\alpha }_{21}{S}_{1}+{\alpha }_{22}{S}_{2}+{\alpha }_{23}H){S}_{2}-{\mu }_{2}{S}_{2};\\ {f}_{3}({S}_{1},{S}_{2},H)={\beta }_{3}({S}_{1},{S}_{2},H)-({\alpha }_{31}{S}_{1}+{\alpha }_{32}{S}_{2}+{\alpha }_{33}H)H-{\mu }_{3}H\mathrm{.}\end{array}$$

The first two functions describe the dynamics of the two sexual forms *S*_1_, *S*_2_ while the last represents that of the asexual hybrid form *H*. We assume that all coefficients are nonnegative; parameters *α*_*ij*_, *i*, *j* = 1, 2, 3, represent intraspecific (*i* = *j*) and interspecific (*i* ≠ *j*) competition (*α*_*ij*_ indicates the effect of species *j* on species *i*) and *μ*_1_, *μ*_2_, *μ*_3_ are mortality coefficients (see^40^).

The growth of respective sexual or asexual forms are described by the birth functions *β*_1_, *β*_2_ and *β*_3_. In order to test the stability of our conclusions against various biological assumptions, we incorporated several types of population birth functions.

The first type of growth functions *β*_*i*_ are of the form as adapted from^42^. These functions describe the growth of populations conditional on the probabilities of the formation of inter- and intraspecific mating couples of males and females while taking into account the mating preferences. In contrast to^42^, where a choice of females for specific males was studied, in current paper we describe the opposite choice, i.e. the choice of males for specific females.

2 $$\begin{array}{rcl}{\beta }_{1}({S}_{1},{S}_{2},H) & = & \frac{\frac{{S}_{1}^{\male}}{2}\frac{{S}_{1}^{\female}}{2}}{\frac{{S}_{1}^{\female}}{2}+A(\frac{{S}_{2}^{\female}}{2}+H)};\\ {\beta }_{2}({S}_{1},{S}_{2},H) & = & \frac{\frac{{S}_{2}^{\male}}{2}\frac{{S}_{2}^{\female}}{2}}{\frac{{S}_{2}^{\female}}{2}+B(\frac{{S}_{1}^{\female}}{2}+H)};\\ {\beta }_{3}({S}_{1},{S}_{2},H) & = & \frac{A\frac{{S}_{1}^{\male}}{2}(\frac{{S}_{2}^{\female}}{2}+H)}{\frac{{S}_{1}^{\female}}{2}+A(\frac{{S}_{2}^{\female}}{2}+H)}+\frac{B\frac{{S}_{2}^{\male}}{2}(\frac{{S}_{1}^{\female}}{2}+H)}{\frac{{S}_{2}^{\female}}{2}+B(\frac{{S}_{1}^{\female}}{2}+H)}\mathrm{.}\end{array}$$

We followed^42^ to implement the mating preferences of the males of sexual form *S*_1_ and *S*_2_, for the females of other forms (i.e. of the other species or pseudogamous individuals) relative to their conspecifics. For the sake of simplicity, we assume that the males have the same preference for other sexual females and asexual ones. The male mating preferences are thus controlled by the parameters *A* and *B*, so that *A* = 0 means that the males of the first species (${S}_{1}^{\male}\mathrm{/2}$) exclusively prefer their own females and do not interact with females of the second species (${S}_{2}^{\female}\mathrm{/2}$) nor with asexuals (*H*), whereas *A* = 1 means that these males do not distinguish between any forms of female at all. Parameter *B* has the same meaning for the males of the second species (*S*_2_). The case *A* = *B* = 0 represents the situation where the males completely recognize and prefer exclusively their own females and no hybrids can appear.

Given that the offspring of the first sexual species may recruit only from the mating of conspecific parents, the fraction $\frac{{S}_{1}^{\female}\mathrm{/2}}{{S}_{1}^{\female}\mathrm{/2}+A({S}_{2}^{\female}\mathrm{/2}+H)}$ in function *β*_1_ determines the ratio of own females, i.e. of females of the first species (${S}_{1}^{\female}\mathrm{/2}$) to the number of all ${S}_{1}^{\female}\mathrm{/2}+A({S}_{2}^{\female}\mathrm{/2}+H)$ females that are available for the males of the first species. This ratio depends on the parameter *A*, c.f.^42^. Similar considerations lead to the form of *β*_2_. The form of the function *β*_3_ reflects the fact that hybrids can be born only by mating the ${S}_{1}^{\male}$-form or ${S}_{2}^{\male}$-form males with heterospecific females (of the other species or pseudogams), i.e. by mating ${S}_{1}^{\male}\mathrm{/2}$ males with the favorable ratio $A\frac{\frac{{S}_{2}^{\female}}{2}+H}{\frac{{S}_{1}^{\female}}{2}+A(\frac{{S}_{2}^{\female}}{2}+H)}$ of females of the second species and of asexual hybrids or vice versa.

This form of birth function has an undesirable property of strong limitation of the inferior sex (in our case the model is male-dominated since females, including the hybrid ones, may form dominant component of simulated populations, thereby leaving only small fraction of ‘free’ males). We therefore also used the modified birth functions implementing the harmonical means, which enforce the intermediate dominance and are used in modelling the population with distinct sexes (see e.g.^43^ [Section F, Eqn.(25.26)]). Then these functions are of the form

3 $$\begin{array}{rcl}{\beta }_{1}({S}_{1},{S}_{2},H) & = & \frac{2\frac{{S}_{1}^{\male}}{2}\frac{{S}_{1}^{\female}}{2}}{\frac{{S}_{1}^{\male}}{2}+\frac{{S}_{1}^{\female}}{2}+A(\frac{{S}_{2}^{\female}}{2}+H)};\\ {\beta }_{2}({S}_{1},{S}_{2},H) & = & \frac{2\frac{{S}_{2}^{\male}}{2}\frac{{S}_{2}^{\female}}{2}}{\frac{{S}_{2}^{\male}}{2}+\frac{{S}_{2}^{\female}}{2}+B(\frac{{S}_{1}^{\female}}{2}+H)};\\ {\beta }_{3}({S}_{1},{S}_{2},H) & = & \frac{2A\frac{{S}_{1}^{\male}}{2}(\frac{{S}_{2}^{\female}}{2}+H)}{\frac{{S}_{1}^{\male}}{2}+\frac{{S}_{1}^{\female}}{2}+A(\frac{{S}_{2}^{\female}}{2}+H)}+\frac{2B\frac{{S}_{2}^{\male}}{2}(\frac{{S}_{1}^{\female}}{2}+H)}{\frac{{S}_{2}^{\male}}{2}+\frac{{S}_{2}^{\female}}{2}+B(\frac{{S}_{1}^{\female}}{2}+H)}\,\mathrm{.}\end{array}$$

Both above described types of *β*_*i*_ growth functions assumes that female and male have identical rates of gamete productions and can mate repeatedly with other male types. To take into account stronger gamete limitation in females, we also implemented a penalizing term to the *β*_*i*_ growth functions modelling a situation when once a female has been inseminated by a male (conspecific or heterospecific), she might have not intercourse again. This assumption has been implemented in the form of cross-link terms reducing the numbers of available females. The birth functions now take the form

4 $$\begin{array}{rcl}{\beta }_{1}({S}_{1},{S}_{2},H) & = & \frac{\frac{{S}_{1}^{\male}}{2}\frac{{S}_{1}^{\female}}{2}}{\frac{{S}_{1}^{\female}}{2}+A(\frac{{S}_{2}^{\female}}{2}+H)}(1-B\frac{{S}_{2}^{\male}}{{S}_{1}^{\male}+{S}_{2}^{\male}});\\ {\beta }_{2}({S}_{1},{S}_{2},H) & = & \frac{\frac{{S}_{2}^{\male}}{2}\frac{{S}_{2}^{\female}}{2}}{\frac{{S}_{2}^{\female}}{2}+B(\frac{{S}_{1}^{\female}}{2}+H)}(1-A\frac{{S}_{1}^{\male}}{{S}_{1}^{\male}+{S}_{2}^{\male}});\\ {\beta }_{3}({S}_{1},{S}_{2},H) & = & \frac{A\frac{{S}_{1}^{\male}}{2}(\frac{{S}_{2}^{\female}}{2}+H)}{\frac{{S}_{1}^{\female}}{2}+A(\frac{{S}_{2}^{\female}}{2}+H)}+\frac{B\frac{{S}_{1}^{\male}}{2}(\frac{{S}_{1}^{\female}}{2}+H)}{\frac{{S}_{2}^{\female}}{2}+B(\frac{{S}_{1}^{\female}}{2}+H)}\mathrm{.}\end{array}$$

and

5 $$\begin{array}{rcl}{\beta }_{1}({S}_{1},{S}_{2},H) & = & \frac{2\frac{{S}_{1}^{\male}}{2}\frac{{S}_{1}^{\female}}{2}}{\frac{{S}_{1}^{\male}}{2}+\frac{{S}_{1}^{\female}}{2}+A(\frac{{S}_{2}^{\female}}{2}+H)}(1-B\frac{{S}_{2}^{\male}}{{S}_{1}^{\male}+{S}_{2}^{\male}});\\ {\beta }_{2}({S}_{1},{S}_{2},H) & = & \frac{2\frac{{S}_{2}^{\male}}{2}\frac{{S}_{2}^{\female}}{2}}{\frac{{S}_{2}^{\male}}{2}+\frac{{S}_{2}^{\female}}{2}+B(\frac{{S}_{1}^{\female}}{2}+H)}(1-A\frac{{S}_{1}^{\male}}{{S}_{1}^{\male}+{S}_{2}^{\male}});\\ {\beta }_{3}({S}_{1},{S}_{2},H) & = & \frac{2A\frac{{S}_{1}^{\male}}{2}(\frac{{S}_{2}^{\female}}{2}+H)}{\frac{{S}_{1}^{\male}}{2}+\frac{{S}_{1}^{\female}}{2}+A(\frac{{S}_{2}^{\female}}{2}+H)}+\frac{2B\frac{{S}_{2}^{\male}}{2}(\frac{{S}_{1}^{\female}}{2}+H)}{\frac{{S}_{2}^{\male}}{2}+\frac{{S}_{2}^{\female}}{2}+B(\frac{{S}_{1}^{\female}}{2}+H)}\,,\end{array}$$

respectively.

Here, the number ${S}_{1}^{\female}\mathrm{/2}$ of females of the first species that are available for their own males is reduced by those that have spawned with heterospecific males. The fraction of ‘occupied’ females of the first species is thus proportional to the proportion of the ${S}_{1}^{\male}\mathrm{/2}$ males in the total male population scaled by the factor of their preference for conspecifics (B). This fraction thus takes the form $B\frac{{S}_{2}^{\male}\mathrm{/2}}{({S}_{1}^{\male}\mathrm{/2}+{S}_{2}^{\male}\mathrm{/2)}}$ and hence, the fraction of ‘free’/available females becomes $(1-B\frac{{S}_{2}^{\male}\mathrm{/2}}{{S}_{1}^{\male}\mathrm{/2}+{S}_{2}^{\male}\mathrm{/2}})$. There is no corresponding reduction of ‘free’ males since we assume no limitations for male mating. Also, we employ no such reduction for the hybrid females since all types of heterospecific mating lead to their population growth.

First, we studied the behavior of the system of ordinary differential equations (ODE), which describe the coexistence of the three forms in time

6 $$\begin{array}{l}\frac{\,{\rm{d}}}{\,{\rm{d}}t}{S}_{1}={f}_{1}({S}_{1},{S}_{2},H),\\ \frac{\,{\rm{d}}}{\,{\rm{d}}t}{S}_{2}={f}_{2}({S}_{1},{S}_{2},H),\\ \frac{\,{\rm{d}}}{\,{\rm{d}}t}H={f}_{3}({S}_{1},{S}_{2},H\mathrm{).}\end{array}$$

Subsequently, we investigated the behavior of such a system in both time and space by simulating the dispersal of all three forms on a single-dimensional space. For this purpose, we used the system of partial differential equations (PDE) of reaction-diffusion type

7 $$\begin{array}{l}{\partial }_{t}{S}_{1}={d}_{1}{\partial }_{xx}{S}_{1}+{f}_{1}({S}_{1},{S}_{2},H),\\ {\partial }_{t}{S}_{2}={d}_{2}{\partial }_{xx}{S}_{2}+{f}_{2}({S}_{1},{S}_{2},H),\\ {\partial }_{t}H={d}_{3}{\partial }_{xx}H+{f}_{3}({S}_{1},{S}_{2},H\mathrm{).}\end{array}$$

The spatial distributions of the three forms are given by the parameters *d*_1_, *d*_2_ and *d*_3_ corresponding to the diffusion speeds of *S*_1_, *S*_2_, and *H*. For simplicity and numerical tractability, our models assume spatial expansion in one-dimensional space. Although such a simplification may omit some outcomes, which may appear in 2-D models^32^, it greatly facilitates the model analysis and is still likely to capture the key features^41^ (p.439)). Each simulation was initiated by introducing *S*_1_ and *S*_2_ species at opposite ends of the space and letting them disperse until they made contact, when they started to produce pseudogamous hybrid females. Such females may subsequently invade the territories of both sexual species and compete with them for resources and sperm.

### Simulation procedure and parameter values

We did not search for analytical solutions since they are very difficult to obtain in a closed form for nonlinear higher-order LV systems (e.g.^37^), and it is impossible for such systems with a spatial diffusion,. Instead, we used numerical bifurcation analysis to investigate the behavior of the above systems of ODE as well as PDE. For ODE, at *t* = 0 we initially set *H* = 0 and *S*_1_ = *S*_2_ to various initial densities (see the Results section).

For PDE we considered zero flux (i.e. Neumann) boundary conditions for all variables. In the numerical analysis we firstly introduced both sexual species at opposite extremities of the domain along a linear space interval. The initial densities of both sexual species and hybrid were set to zero at all points except the two left and right extremities, where *S*_1_ and *S*_2_ species were implemented at arbitrarily chosen density (see the Results section). To evaluate the effects of initial conditions on performed simulations, we ran the same simulations also with different initial seed values for both sexual populations (relative initial proportions of *S*_1_ to *S*_2_ varied from 0.01 to 100). The full simulation strategy is shown in Supplementary Table ST1.

### Asymmetries in sensitivity of sexual species to pseudogams and types of simulation runs

Behavior of the sexual-pseudogamous complex has been studied by changing the values of competitive parameters and relative values of *A* and *B* coefficients (i.e. male mating preferences). For the numerical analysis of our model, we first set all parameters equal for all populations. (*d*_*i*_ = 0.01, *α*_*ij*_ = 1, *μ*_1_ = *μ*_2_ = *μ*_3_ = 0.2, *A* = *B* = 1 for models given by Eqs (2) and (4) and *d*_*i*_ = 0.02, *α*_*ij*_ = 1, *μ*_1_ = *μ*_2_ = *μ*_3_ = 0.1, *A* = *B* = 1 for models given by Eqs (3) and (5)).

To evaluate the effect of pseudogams on the competition and hybridization between two sexual species, which differ in ‘resistance’ to such sperm-parasites, we investigated two sorts of asymmetries.

*Asymmetry type 1*: We treated *S*_1_ and *S*_2_ species as differing in male mating preferences (hence, we kept *A* constant and varied *B* from 1 to 0.1).

*Asymmetry type 2*: We suggested that the presence of pseudogams exerted asymmetrical competitive pressure on both sexual species *S*_1_ and *S*_2_ and so kept *α*_13_ constant and varied *α*_23_ from 0.2 to 2.

The biological properties of pseudogamous systems present possible specific problems that were addressed in our models. The first case is that if all else is equal among sexual and asexual forms, asexuals tend to outcompete their sexual counterparts due to intrinsic reproductive advantage^44^, which leads to the collapse of the whole system. To prevent such behavior and to ensure that at least one sexual species usually persist until the end of simulation, we investigated the competitions under two different forms of stabilizing mechanisms allowing the coexistence of sexual and sperm-dependent asexual forms.

*Stabilizing Mechanism SM1*^44^: proposed that asexuals are limited by a higher intensity of intraspecific competition because clonal individuals face more intensive competition with their siblings since clonally propagated genotypes are more similar to each other than sexually propagated ones. We have therefore increased the parameter *α*_33_, while keeping the remaining coefficients of intraspecific competition *α*_*ii*_ = 1.

*Stabilizing Mechanism SM2*: we conjecture as in e.g.^42^ that male mate choice is the stabilizing force and all species are able to discriminate between their own females and other females including asexual hybrids, therefore balancing the reproductive advantage of asexual females. In this case, we assumed that asexuals were identical to sexuals in all parameters (*α*_*ii*_ = 1) and set the mate-recognition parameter *A* = 0.5 (the values of *B* varied as we investigated their influence on the model behavior).

The second complication is that natural pseudogamous asexuals are mostly of hybrid origin (as in our simulation) and hence we had to solve the problem of intrinsic differences between asexuals of hybrid origins compared to their nonhybrid sexual ancestors. Therefore, three types of hybrids were considered.

*Hybrid type 1:* the competitive strength of hybrids was set equal to the superior sexual competitor (*α*_32_ = *α*_12_),

*Hybrid type 2:* the competitive strength of hybrids was set equal to inferior competitor (*α*_13_ = *α*_12_),

*Hybrid type 3:* the competitive strength of hybrids was set to the geometric average of both sexual species (${\alpha }_{13}={\alpha }_{32}=\sqrt{{\alpha }_{12}}$).

(Let us note that the aforementioned definition of hybrid types is relevant only to *asymmetry type 1*).

For each combination of types of stabilizing mechanism, of hybrid type and of the growth function, we performed many simulations with *α*_12_ varying in the range (0.001–1) to simulate the effect of competitive asymmetry and with *B* or *α*_23_ varying in the range (0.1–1) and (0.2–2), respectively, to simulate the effect of asymmetries in ‘resistance’ to shared sperm-parasites.

### Evaluating the magnitude of the effect of hybrid pseudogamy on interspecific competition

To evaluate the extent to which the results are affected by implicit effects of pseudogamy, we compared situations when species compete but do not produce asexual hybrids. To do so, we performed three types of simulations at any point of the parameter space explored. In run type 1, we ran the simulation using the full three-population model (i.e. two sexual species and a pseudogamous hybrid), including all three components of Eqn. (7) as described above.

In run type 2 we also assumed a three-populations model, where both sexual species reproductively interact but produce only sterile hybrids (the hybrid population competes for resources but cannot reproduce). This allows us to compare the effect of pseudogamous hybrids with the effect of the ‘classical’ type of hybridization. Run type 2 represents a special case of a hybrid zone with very strong under-dominance, where competing species reproductively interact, but the fitness of their hybrids was set to zero (extreme under-dominance). We chose this setting because^32^ demonstrated that the formation of a hybrid zone between two competing and hybridizing species forms a barrier between competitors, which tends to slow down the rate of expansion of the superior competitor compared to the pure competition model. Since^32^ showed that the attenuating effect on the hybrid zone advance is greatest with strong under-dominance (see the Discussion section for details), we chose this particular setting as a reference run against which we compared the spatiotemporal dynamics of run type 1.

Finally, we performed the third type of simulation (run type 3) that proposed a pure competition two-population model with no reproductive interference between sexual species.

## Electronic supplementary material

Supplementary Information
