# Supplementary material for: Sperm-dependent asexual hybrids determine competition among sexual species
Source: Sci Rep. 2019 Jan 24;9:722. doi: 10.1038/s41598-018-35167-z (PMC6345890; doi:10.1038/s41598-018-35167-z)
Supplement: Supplementary file 1 — Supplementary Information [file 41598_2018_35167_MOESM1_ESM.pdf]

Sperm-dependent asexual hybrids determine competition among sexual species

Karel Janko , Jan Eisner, Peter Mikulíček

November 29, 2018

Supplementary Material

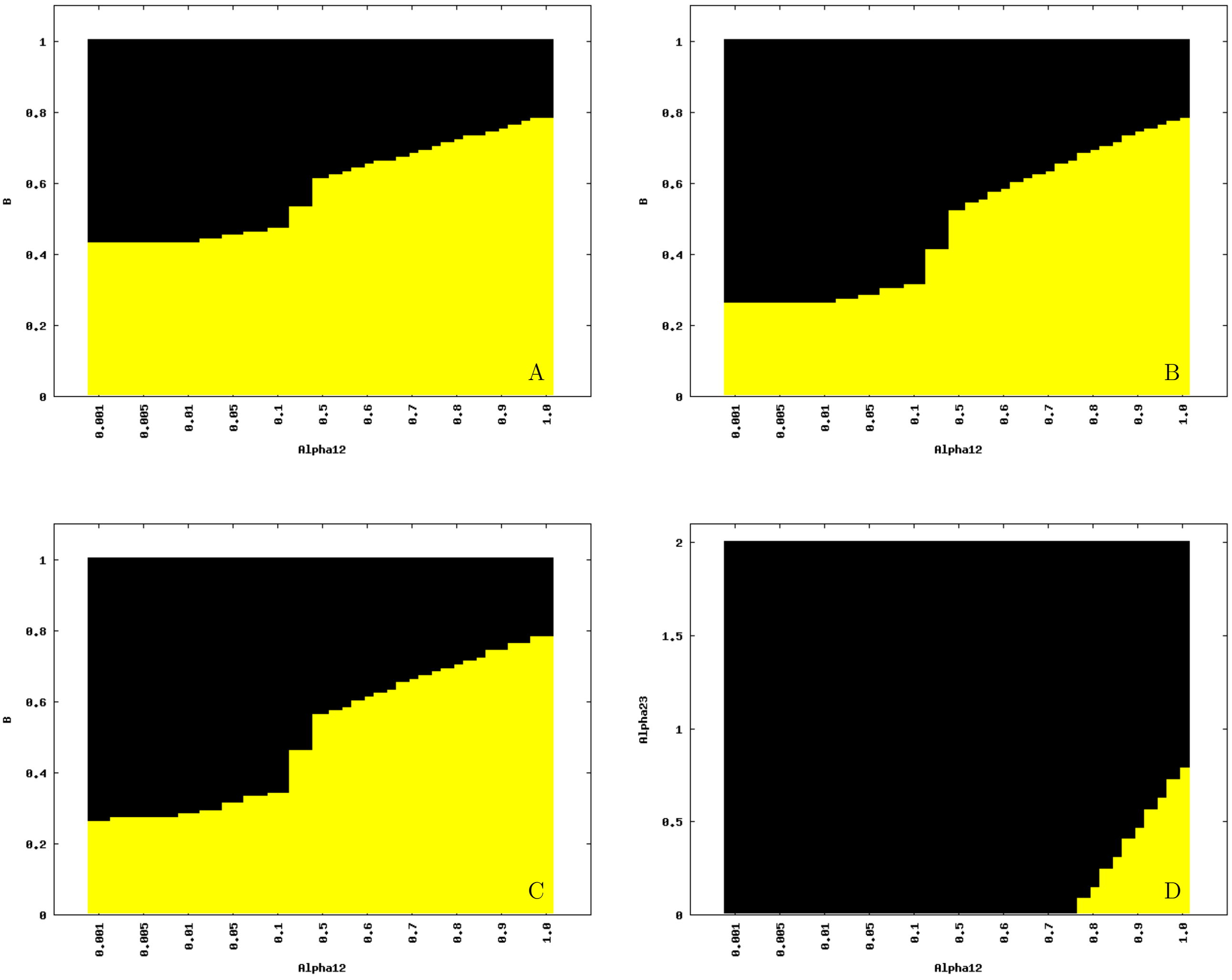

Supplementary Figure SF1: Results of the runs of the ODE systems. Qualitative comparisons of simulations assuming hybridization producing pseudogamous hybrids (run type 1, RT1) with those resulting in hybrid zone formation with strong under-dominance (run type 2, RT2). Panels A–C describe simulations assuming *asymmetry type 1* with hybrids competitively equal to the superior competitor (*hybrid type 1* - panel A), to the inferior competitor (*hybrid type 2* - panel B), or intermediate between both species (*hybrid type 3* - panel C). Panel D describes simulations assuming *asymmetry type 2*.

The parameter space is divided into 11×10 grids (competitive asymmetry between S_1 and S_2 species (α_{12}) changes along x-axis while the resistance against pseudogams (mate choice parameter B or competitive interactions α_{23}) changes along y-axis).

Stabilizing Mechanism SM1 is applied ($\alpha_{33} = 15$), growth functions β_i are of the form Eqn. (2). Parameter $A = 1$, B varies along the y-axis on panels A–C but is fixed at $B = 1$ on panel D. All $\alpha_{ij} = 1$ except α_{12}, which is varied along the x-axis on panels A–D, α_{23} varied along y-axis on panel D and $\alpha_{33} = 15$.

In yellow, there are domains of parameter values where the species S_2 survives and the species S_1 is out-competed. In black, there are domains where S_2 dies out.

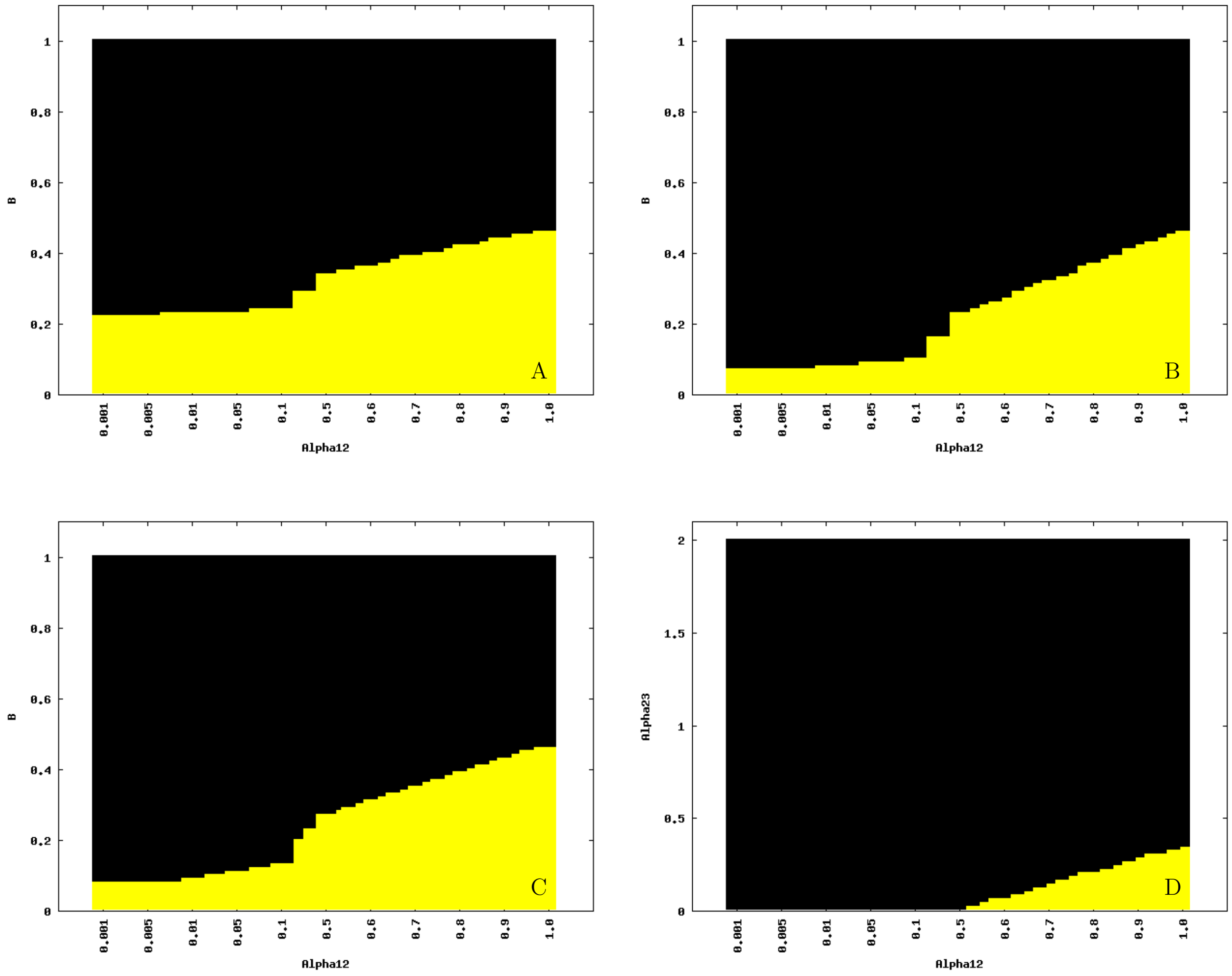

Supplementary Figure SF2: Results of the runs of the ODE systems. Parameters and layout of the panels is the same as on Supplementary Figure SF1. growth functions β_i are of the form Eqn. (2). *Stabilizing Mechanism SM2* is applied ($A = 0.5$). In yellow, the domain where the inferior competitor S_2 out-competes the superior one is visualized in the respective range of parameter values.

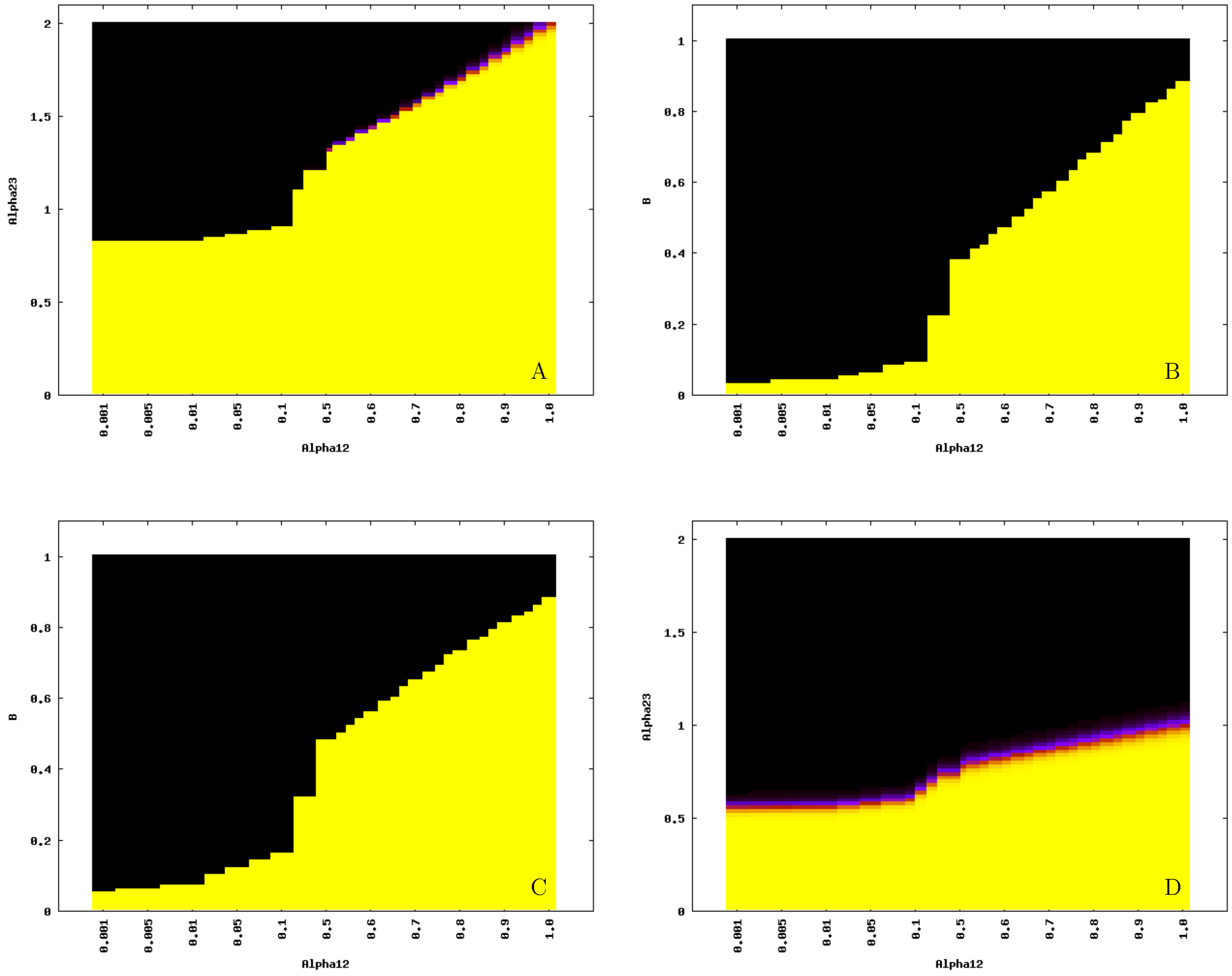

Supplementary Figure SF3: Results of the runs of the ODE systems. Parameters and layout of the panels is the same as on Supplementary Figure SF1. Growth functions β_i are of the form Eqn. (3), *Stabilizing Mechanism SM1* is applied ($\alpha_{33} = 5$).There are 33 (horizontally) times 100 (vertically) calculated grids visualized. In yellow, there are domains of parameter values where the species S_2 survives and the species S_1 is out-competed. In black, there are domains where S_2 dies out.

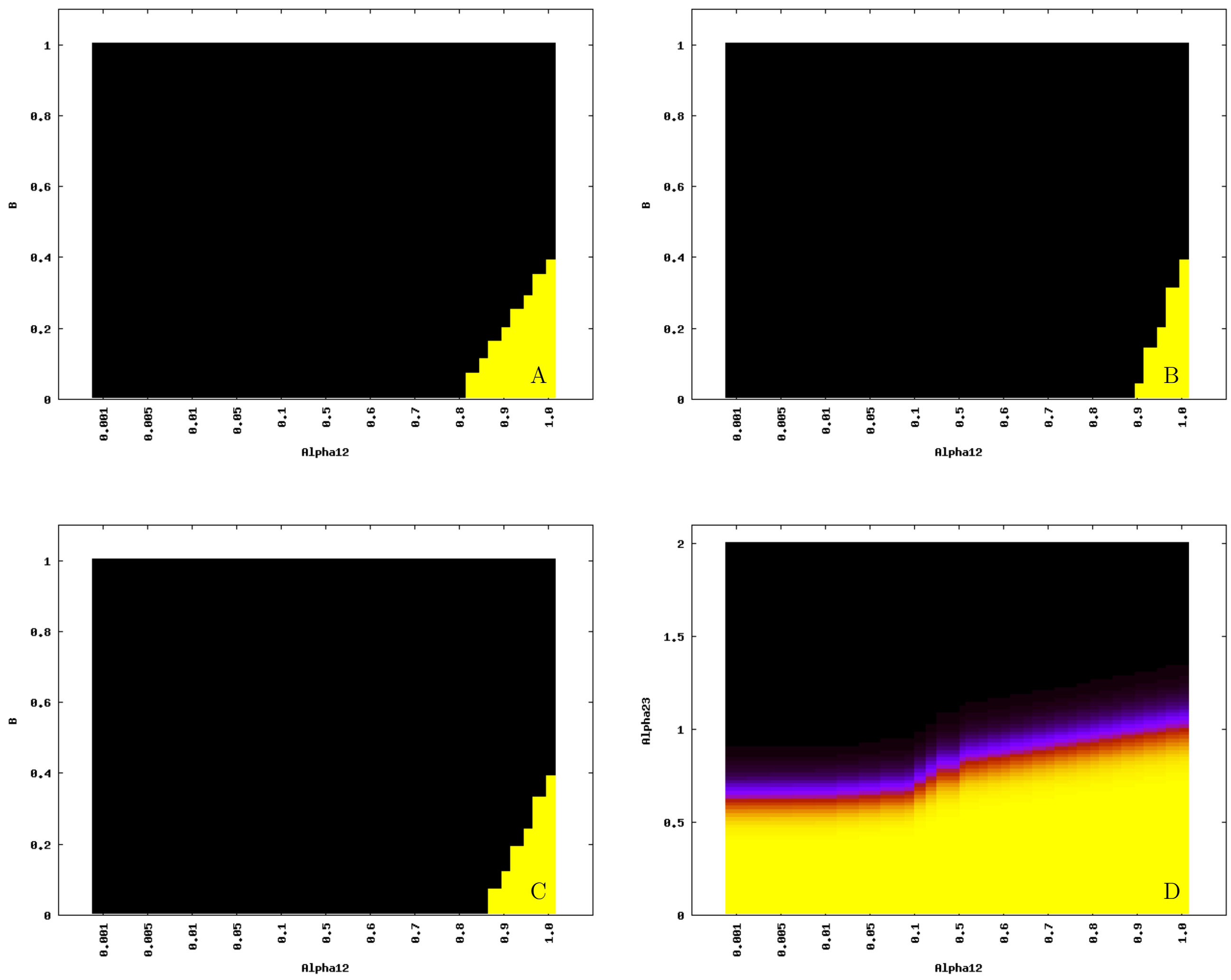

Supplementary Figure SF4: Results of the runs of the ODE systems. Parameters and layout of the panels is the same as on Supplementary Figure SF2. Growth functions β_i are of the form Eqn. (3), *Stabilizing Mechanism SM2* is applied ($A = 0.5$). In yellow, the domain where the inferior competitor S_2 out-competes the superior one is visualized in the respective range of parameter values.

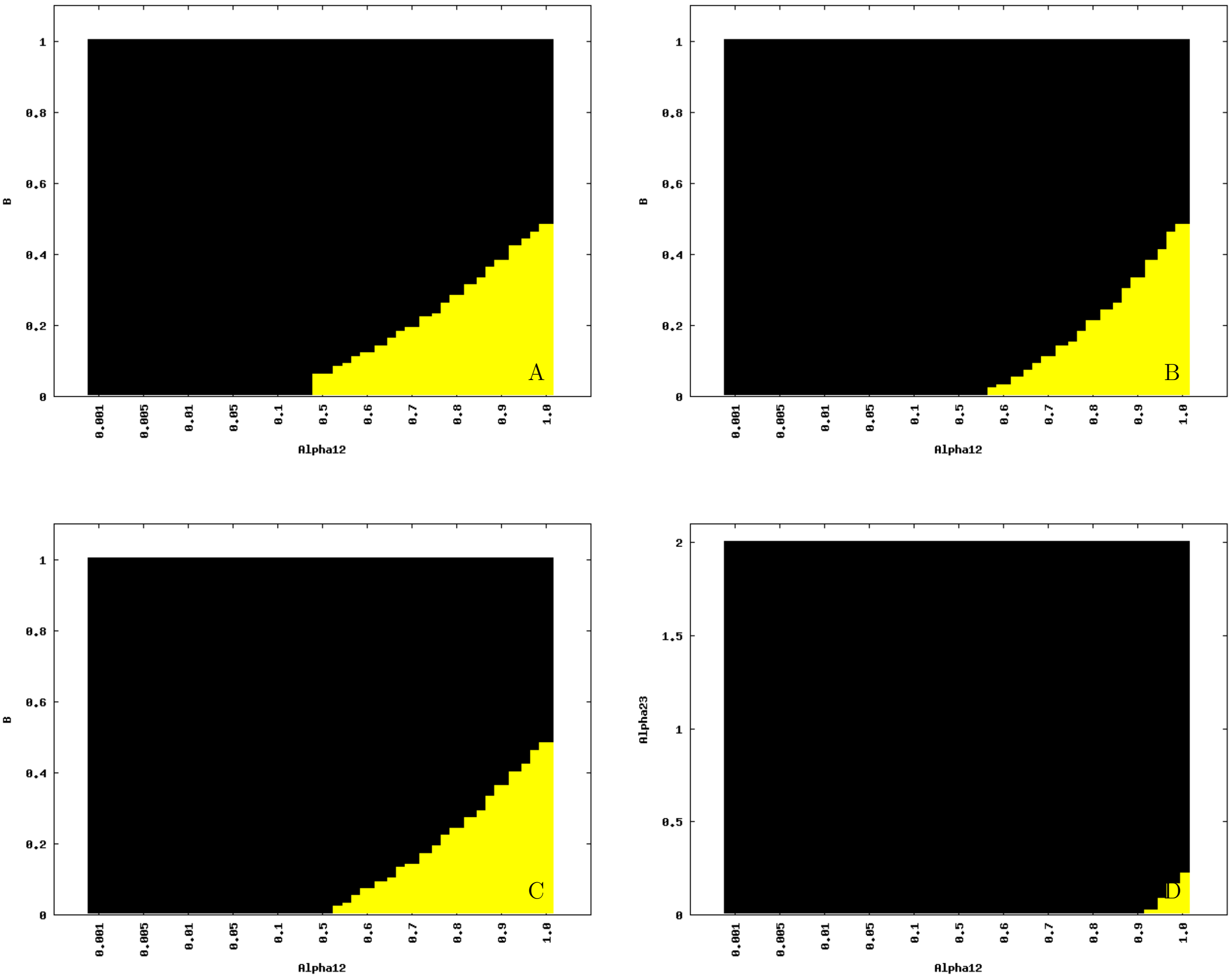

Supplementary Figure SF5: Results of the runs of the ODE systems. Parameters and layout of the panels is the same as on Supplementary Figure SF1. growth functions β_i are of the form Eqn. (4). *Stabilizing Mechanism SM1* is applied ($\alpha_{33} = 15$). In yellow, the domain where the inferior competitor S_2 out-competes the superior one is visualized in the respective range of parameter values.

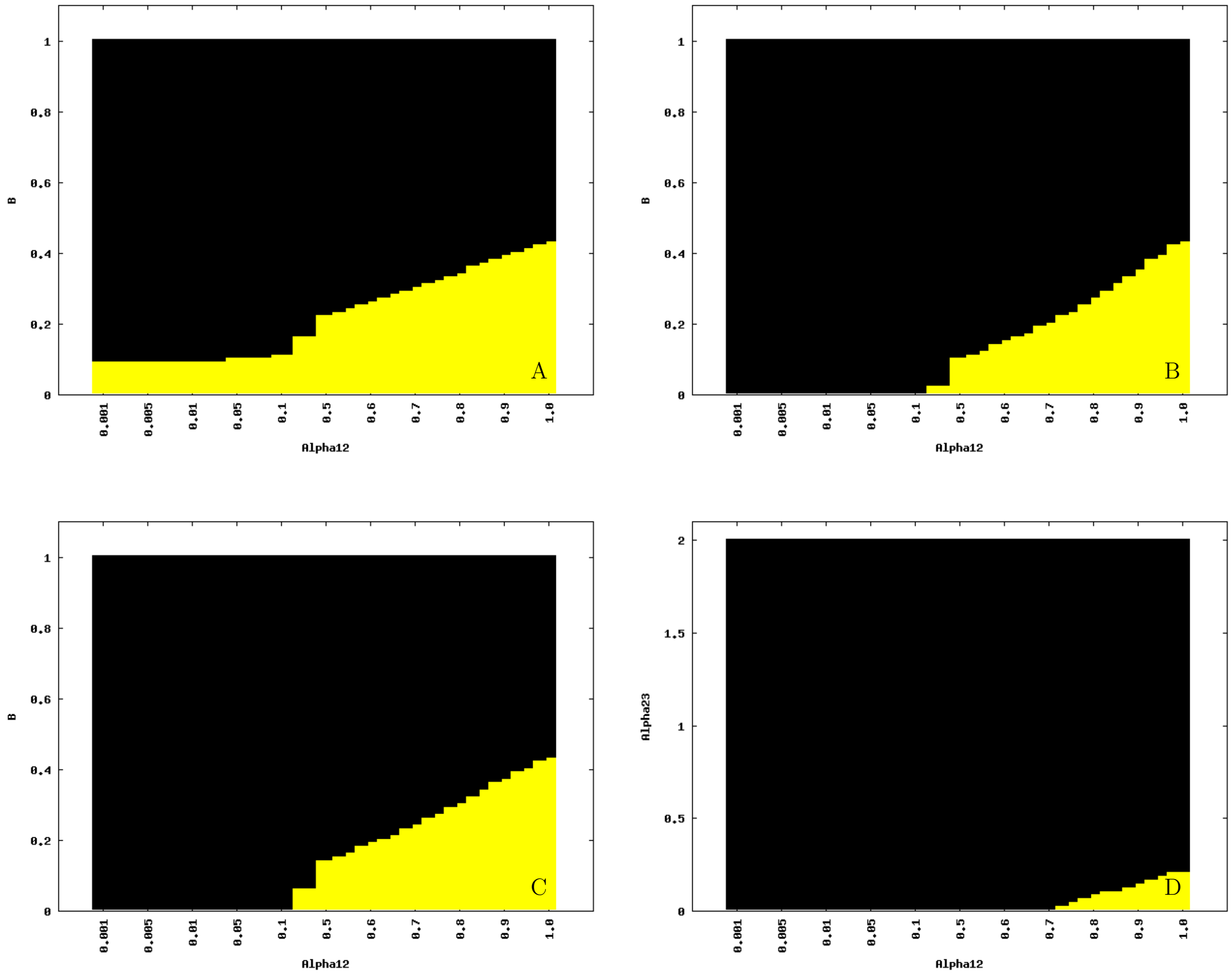

Supplementary Figure SF6: Results of the runs of the ODE systems. Parameters and layout of the panels is the same as on Supplementary Figure SF1. growth functions β_i are of the form Eqn. (4). *Stabilizing Mechanism SM2* is applied ($A = 0.5$). In yellow, the domain where the inferior competitor S_2 out-competes the superior one is visualized in the respective range of parameter values.

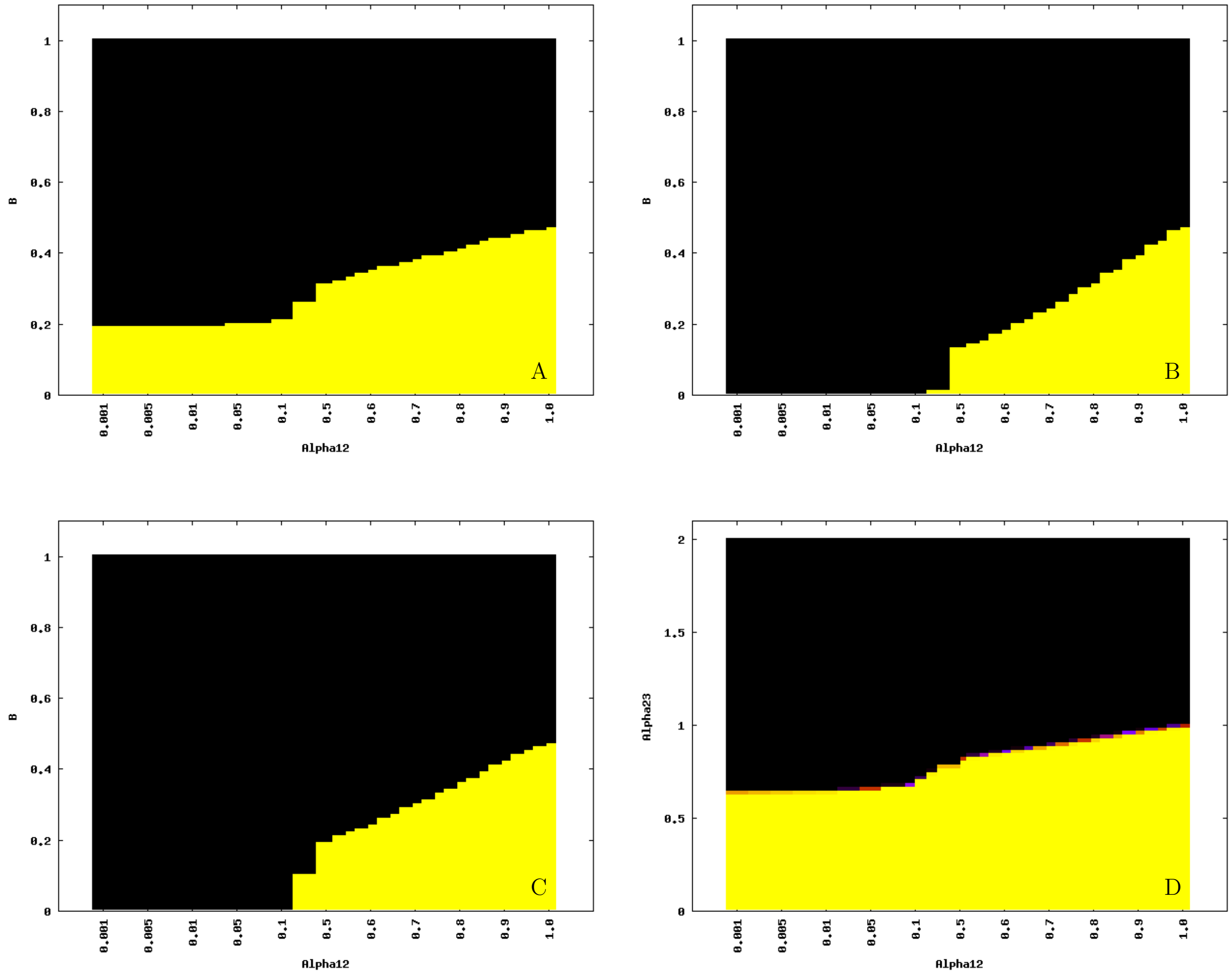

Supplementary Figure SF7: Results of the runs of the ODE systems. Parameters and layout of the panels is the same as on Supplementary Figure SF5. Growth functions β_i are of the form Eqn. (5), *Stabilizing Mechanism SM1* is applied ($\alpha_{33} = 5$). In yellow, the domain where the inferior competitor S_2 out-competes the superior one is visualized in the respective range of parameter values.

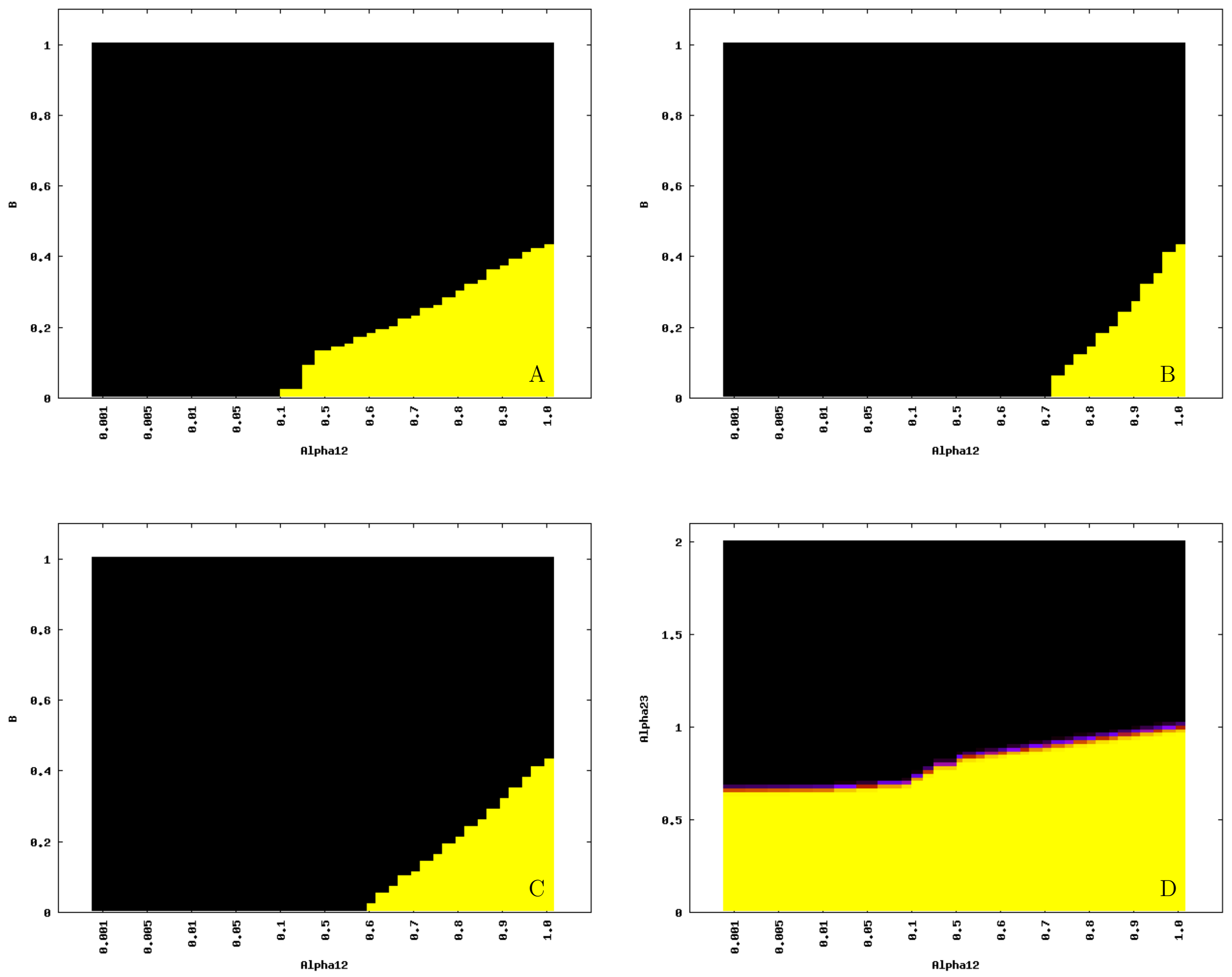

Supplementary Figure SF8: Results of the runs of the ODE systems. Parameters and layout of the panels is the same as on Supplementary Figure SF6. Growth functions β_i are of the form Eqn. (5), *Stabilizing Mechanism SM2* is applied ($A = 0.5$). In yellow, the domain where the inferior competitor S_2 out-competes the superior one is visualized in the respective range of parameter values.

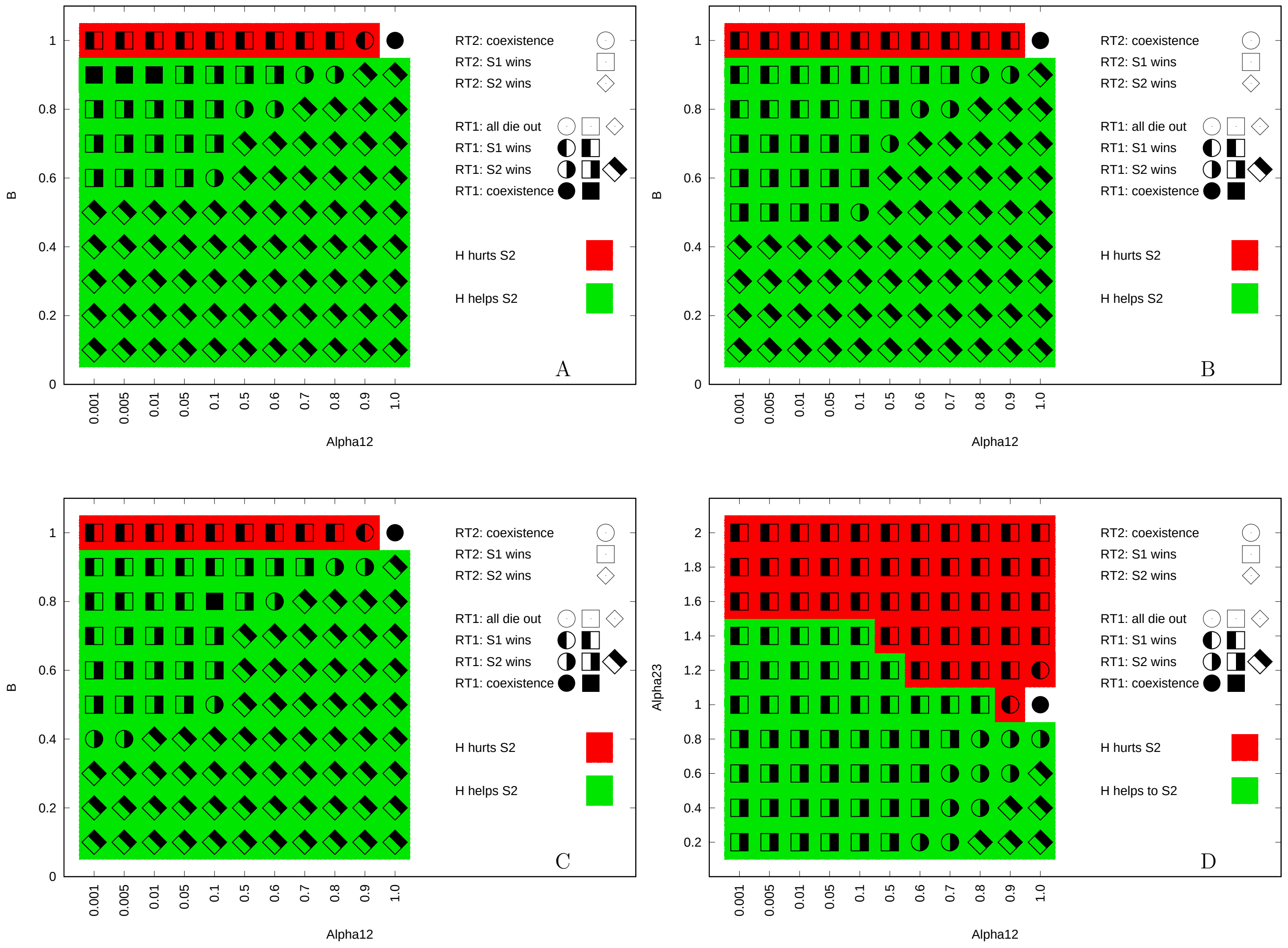

Supplementary Figure SF9: Qualitative comparisons of simulations assuming hybridization producing pseudogamous hybrids (run type 1, RT1) with those resulting in hybrid zone formation with strong under-dominance (run type 2, RT2). Panels A – C describe simulations assuming *asymmetry type 1* with hybrids competitively equal to the superior competitor (*hybrid type 1* - panel A), to the inferior competitor (*hybrid type 2* - panel B), or intermediate between both species (*hybrid type 3* - panel C). Panel D describes simulations assuming *asymmetry type 2*.

The parameter space is divided into 11 × 10 grids (competitive asymmetry between S_1 and S_2 species (α_{12}) changes along x-axis while the resistance against pseudogams (mate choice parameter B or competitive interactions α_{23}) changes along y-axis).

Stabilizing Mechanism SM1 is applied, growth functions β_i are of the form Eqn. (2). Parameter $A = 1$, B varies along the y-axis on panels A–C but is fixed at $B = 1$ on panel D. All $\alpha_{ij} = 1$ except α_{12}, which is varied along the x-axis on panels A–D, α_{23} varied along y-axis on panel D and $\alpha_{33} = 15$.

A meaning of each grid symbol is the same as on Figure 3.

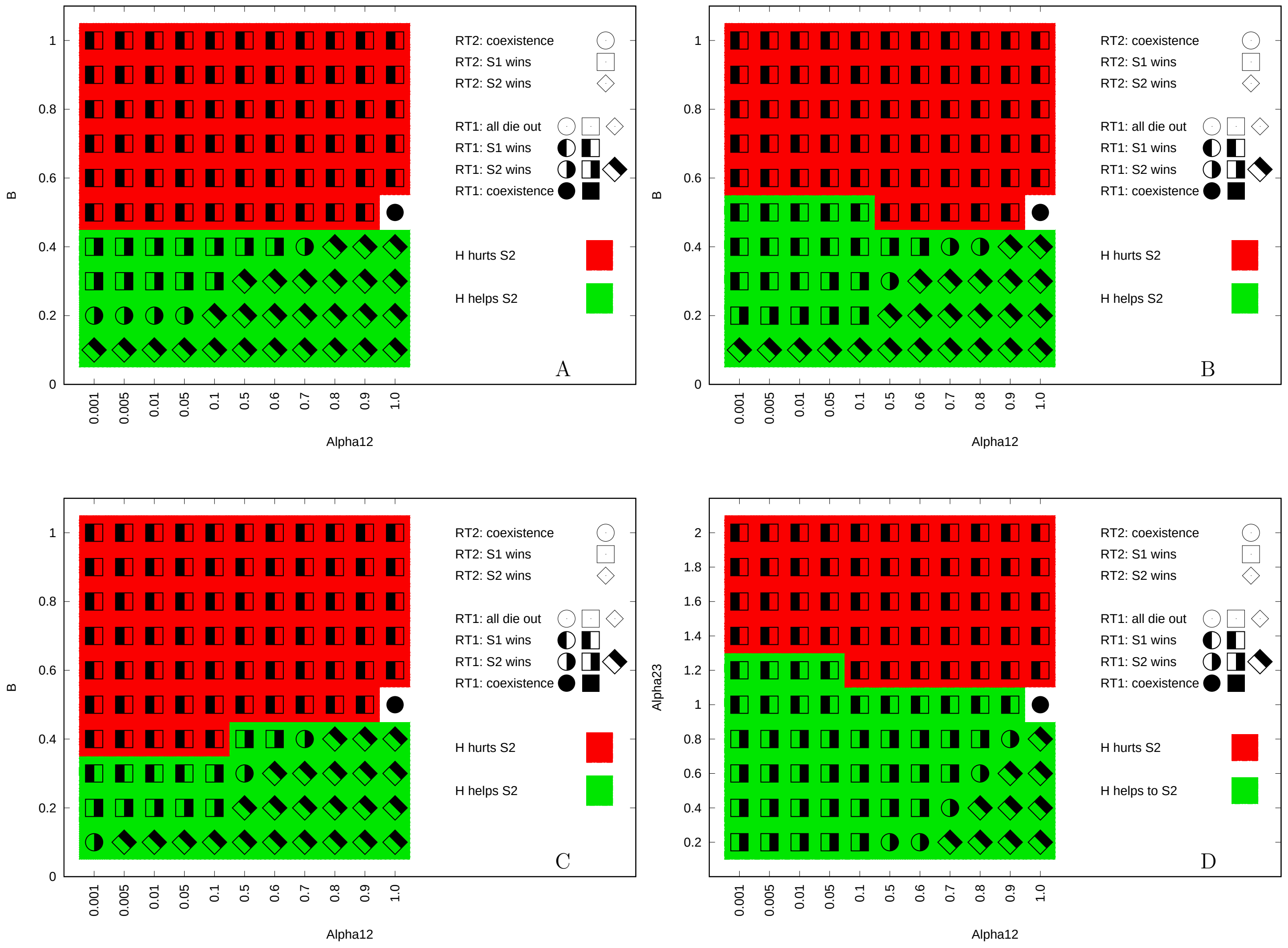

Supplementary Figure SF10: The symbols denote the same as on Supplementary Figure SF9; *Stabilizing Mechanism SM2* is applied, growth functions β_i are of the form Eqn. (2). Parameter values as on Supplementary Figure SF9 except $A = 0.5$, which is used to maintain the stability of the sexual/pseudogamous system (B varies along the y-axis on panels A–C but is fixed at $B = 0.5$ in panel D).

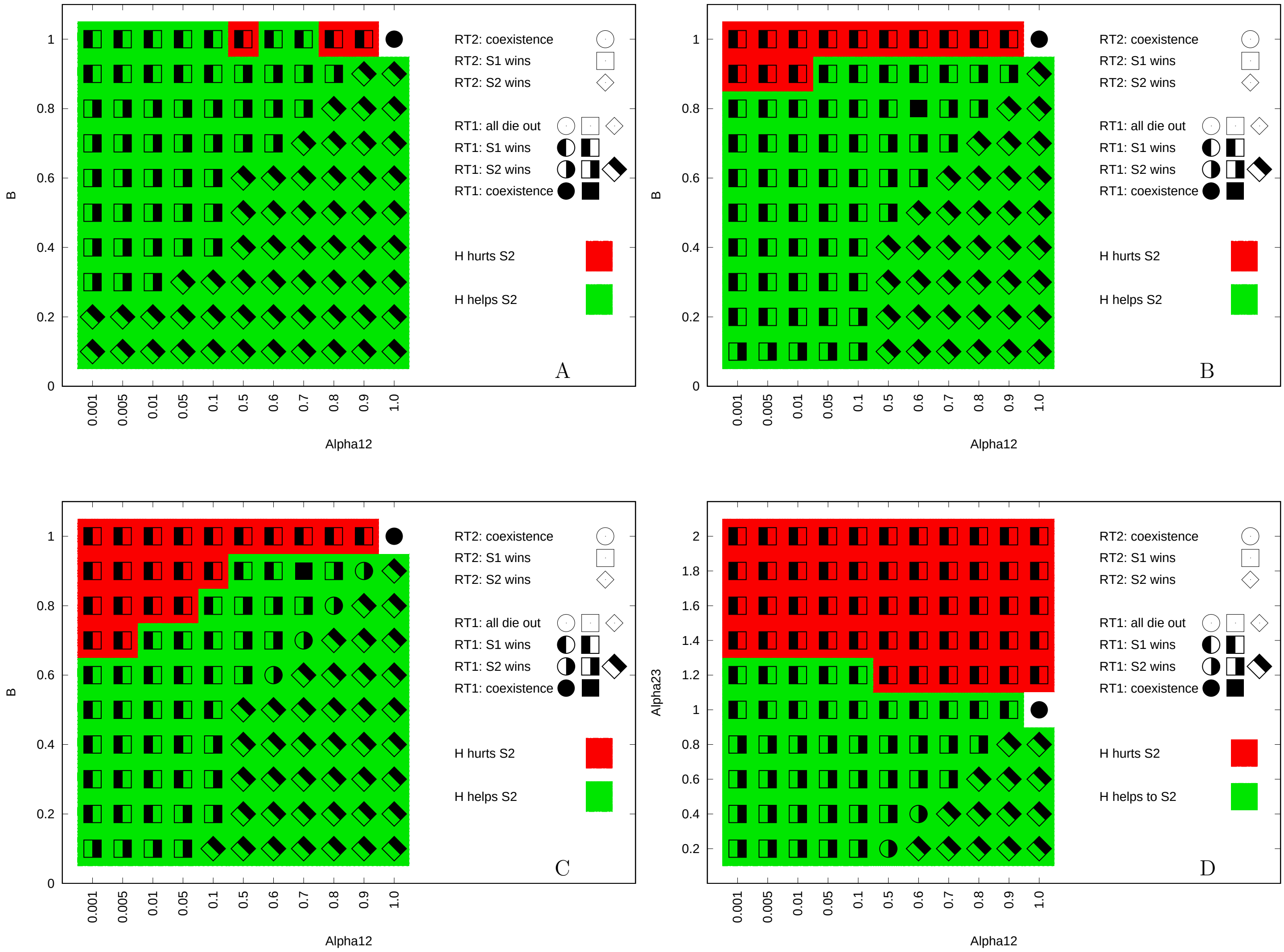

Supplementary Figure SF11: The symbols denote the same as on Supplementary Figure SF9; *Stabilizing Mechanism SM1* is applied, growth functions β_i are of the form Eqn. (3). Parameter values as on Supplementary Figure SF9, $\alpha_{33} = 5$.

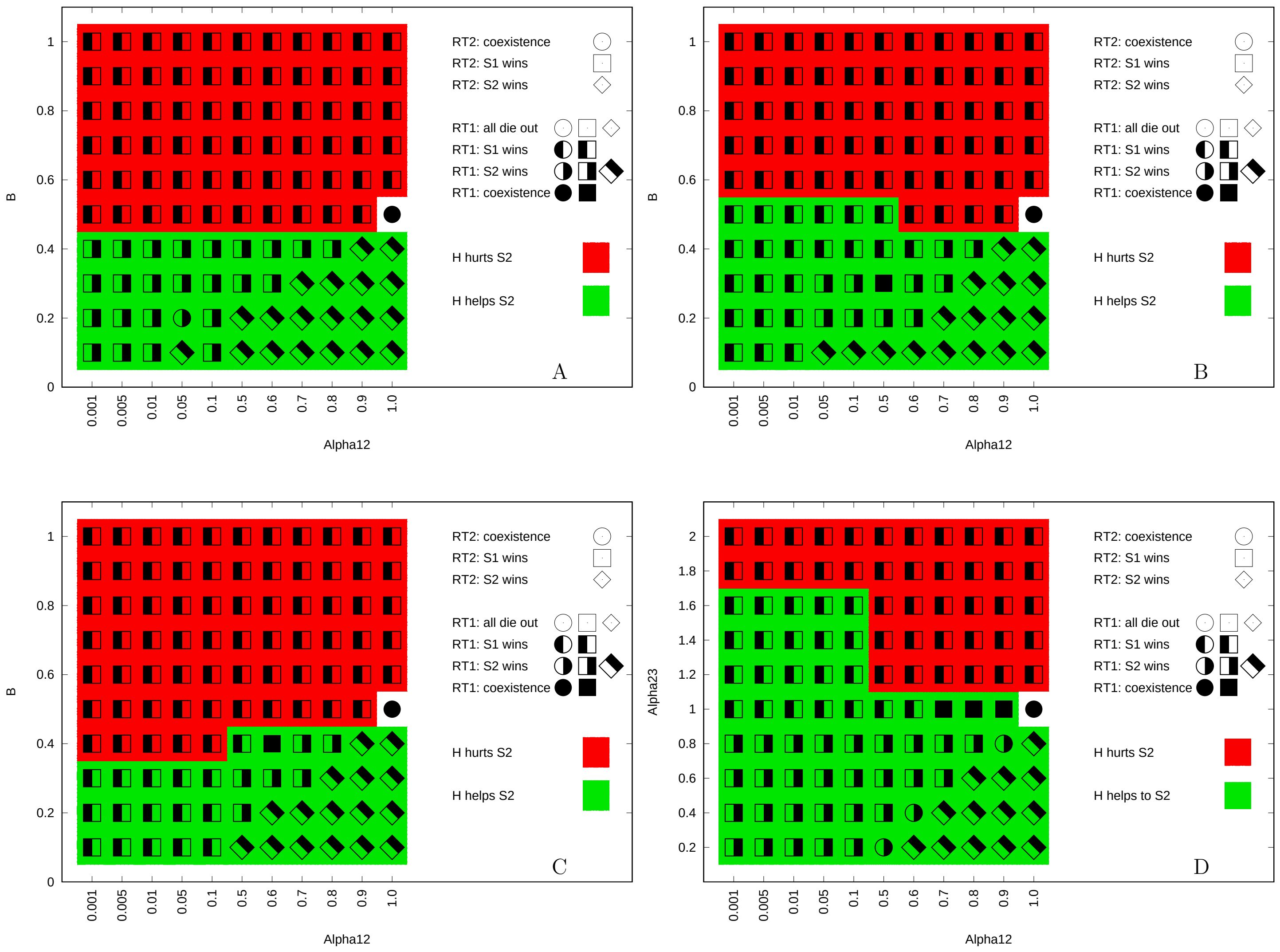

Supplementary Figure SF12: The symbols denote the same as on Supplementary Figure SF9; *Stabilizing Mechanism SM2* is applied, growth functions β_i are of the form Eqn. (3). Parameter values as on Supplementary Figure SF9 except $A = 0.5$, which is used to maintain the stability of the sexual/pseudogamous system (B varies along the y-axis on panels A–C but is fixed at $B = 0.5$ in panel D).

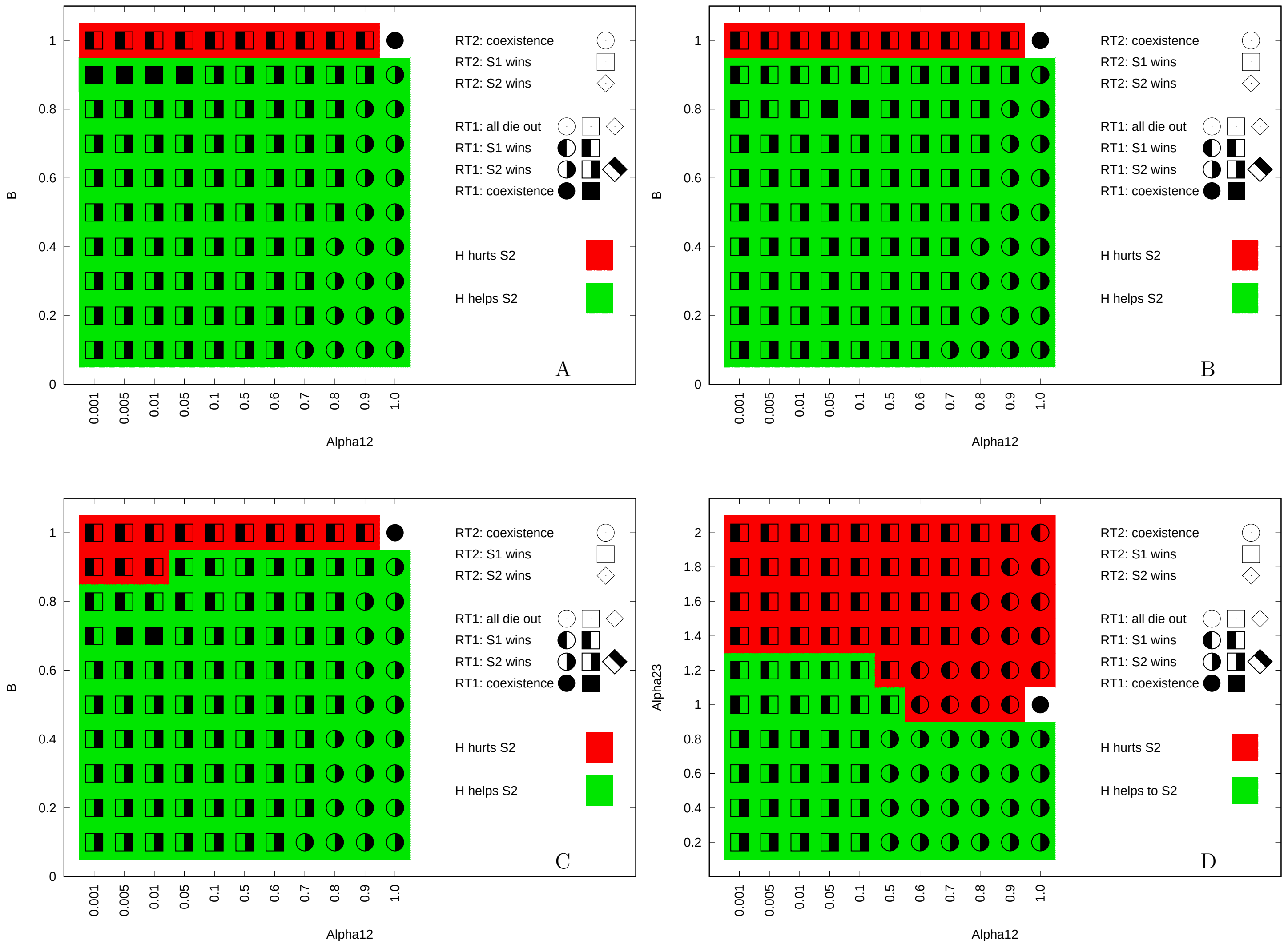

Supplementary Figure SF13: The symbols denote the same as on Supplementary Figure SF9; *Stabilizing Mechanism SM1* is applied, growth functions β_i are of the form Eqn. (4). Parameter values as on Supplementary Figure SF9.

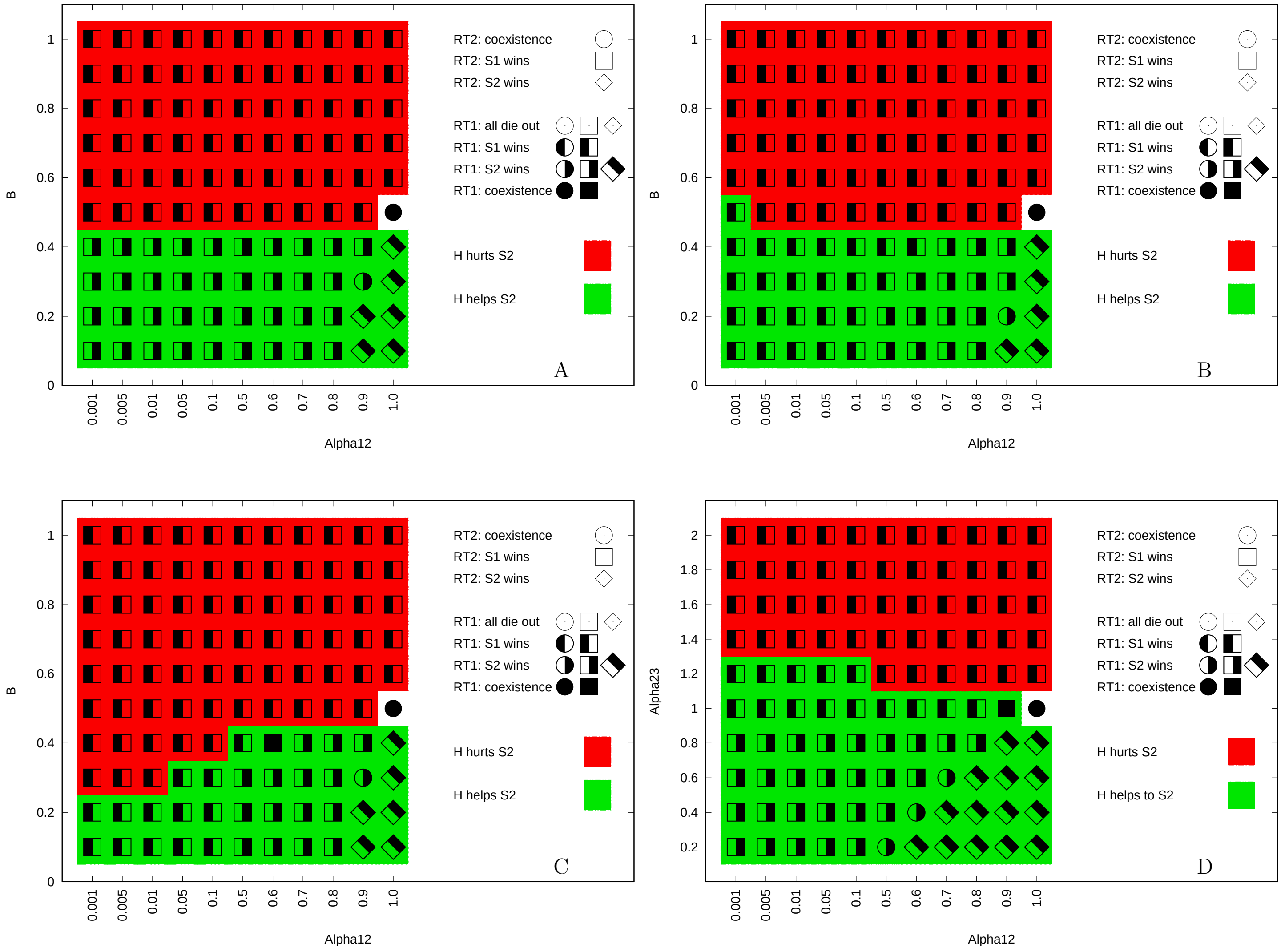

Supplementary Figure SF14: The symbols denote the same as on Supplementary Figure SF9; *Stabilizing Mechanism SM2* is applied, growth functions β_i are of the form Eqn. (4). Parameter values as on Supplementary Figure SF10.

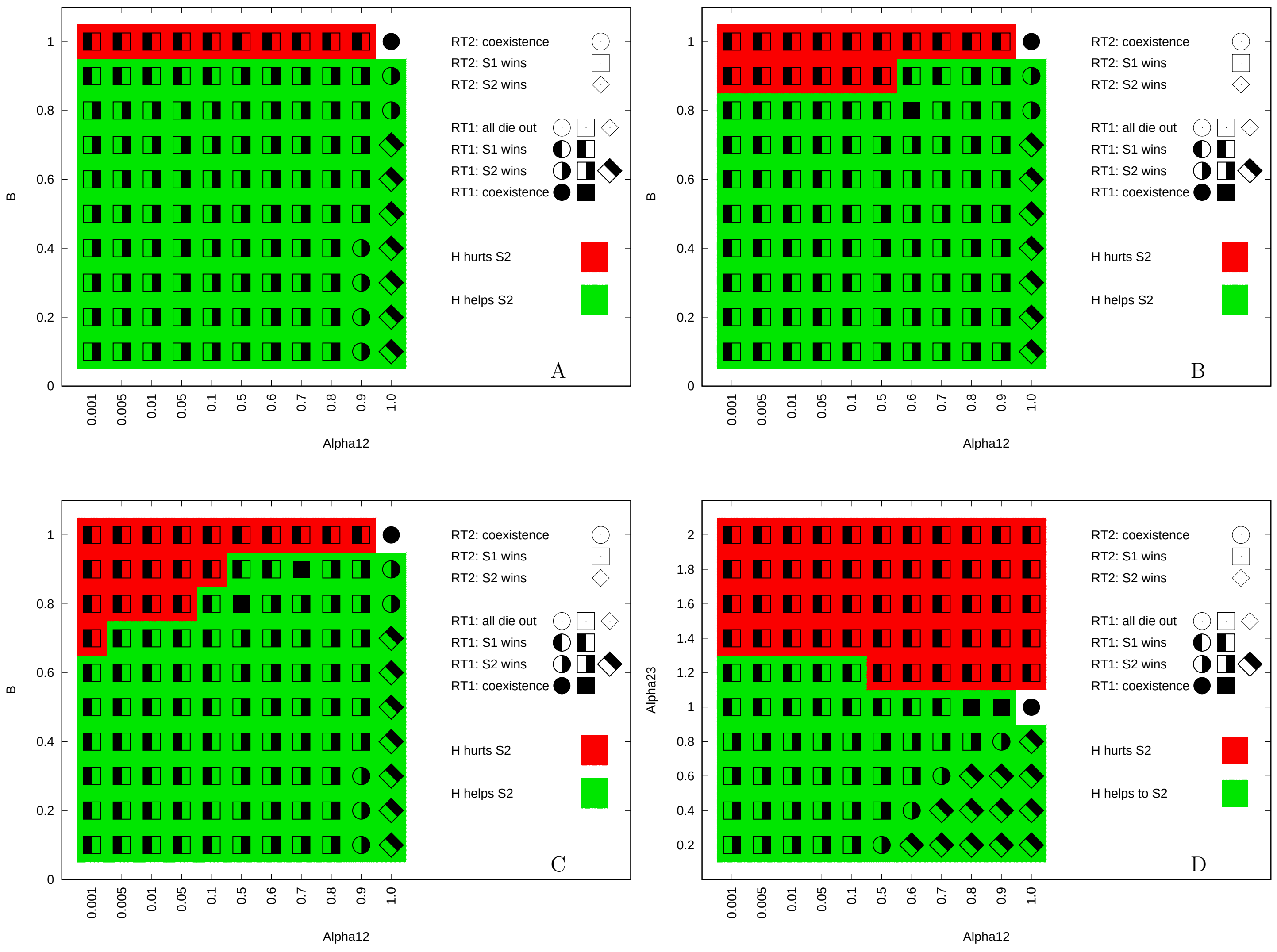

Supplementary Figure SF15: The symbols denote the same as on Supplementary Figure SF9; *Stabilizing Mechanism SM1* is applied, growth functions β_i are of the form Eqn. (5). Parameter values as on Supplementary Figure SF11.

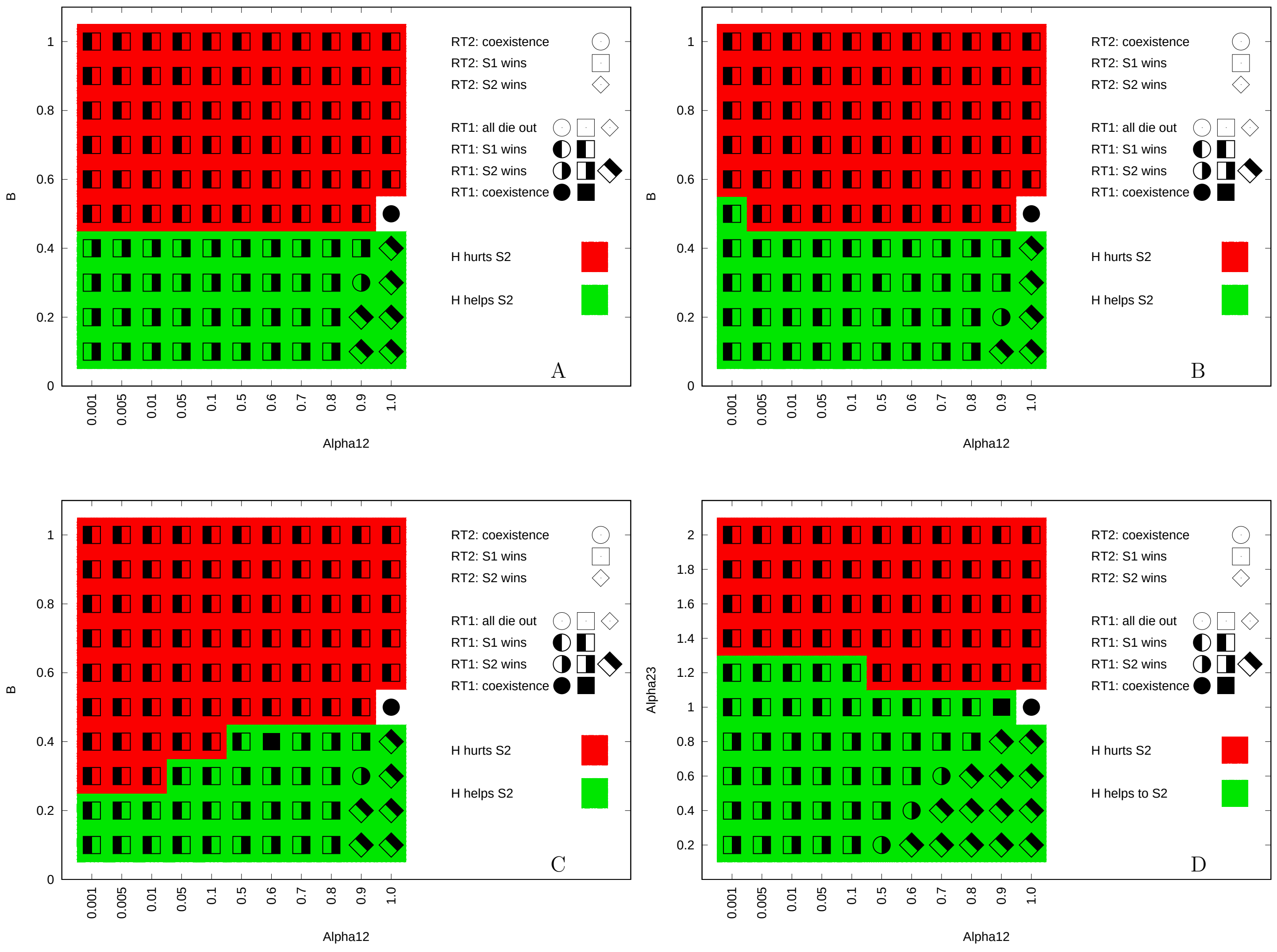

Supplementary Figure SF16: The symbols denote the same as on Supplementary Figure SF9; *Stabilizing Mechanism SM2* is applied, growth functions β_i are of the form Eqn. (5). Parameter values as on Supplementary Figure SF12.

Supplementary Table ST1: Parameters of the models used to evaluate the effect of pseudogamous asexual hybrids to interspecific competition. S_1 and S_2 are two sexual species, H is a pseudogamous hybrid. For detailed explanation of parameters, see Results and Methods.

**Type**		**Parameter**	**Number of values**	**Values**
**Run type**	RT1	full S_1,S_2,H		
	RT2	S_1,S_2,H; H arises by contact of S_1 and S_2 but may not reproduce on its own; no S_1-H, S_2-H matings, only S_1-S_2		
	RT3	Pure competition of S_1,S_2; H does not arise		
**Asymmetry S_1 vs. S_2**	As0	α_{12}	11	0.01—1
	As1	B	10	0.1—1
	As2	α_{23}	10	0.2—2
**Stabilizing mechanism**	SM1	$\alpha_{33} = 15$		
	SM2	A=0.5		
**Hybrid type**	HT1	$\alpha_{32} = \alpha_{12}$		
	HT2	$\alpha_{13} = \alpha_{12}$		
	HT3	$\alpha_{32} = \alpha_{13} = \sqrt{\alpha_{12}}$		
**Growth function**	GF1	aka Som model, unlimited gametes		
	GF2	harmonic mean (Kot model), unlimited gametes		
	GF3	aka Som model, gametes limitations		
	GF4	harmonic mean (Kot model), gametes limitations		
**Initial seed value**	Ini	ratio of initial values of S_1 vs. S_2	10	0.01—100
